# Chitosan oligosaccharide decorated liposomes combined with TH302 for photodynamic therapy in triple negative breast cancer

**DOI:** 10.1186/s12951-021-00891-8

**Published:** 2021-05-19

**Authors:** Yinan Ding, Rui Yang, Weiping Yu, Chunmei Hu, Zhiyuan Zhang, Dongfang Liu, Yanli An, Xihui Wang, Chen He, Peidang Liu, Qiusha Tang, Daozhen Chen

**Affiliations:** 1 Medical School of Southeast University, Nanjing, 210009 China; 2 Research Institute for Reproductive Health and Genetic Diseases, The Affiliated Wuxi Maternity and Child Health Care Hospital of Nanjing Medical University, Wuxi, 214002 China; 3 Department of Tuberculosis, The Second Affiliated Hospital of Southeast University (The Second Hospital of Nanjing), Nanjing, 210009 China; 4 Department of Neurosurgery, Nanjing Jinling Hospital, Nanjing University, Nanjing, 210002 China; 5 Affiliated Zhongda Hospital of Southeast University, Nanjing, 210009 China

**Keywords:** Triple negative breast cancer, Photodynamic therapy, Chitosan oligosaccharide, CD44, Liposomes

## Abstract

**Background:**

Triple negative breast cancer (TNBC) is an aggressive tumor with extremely high mortality that results from its lack of effective therapeutic targets. As an adhesion molecule related to tumorigenesis and tumor metastasis, cluster of differentiation-44 (also known as CD44) is overexpressed in TNBC. Moreover, CD44 can be effectively targeted by a specific hyaluronic acid analog, namely, chitosan oligosaccharide (CO). In this study, a CO-coated liposome was designed, with Photochlor (HPPH) as the 660 nm light mediated photosensitizer and evofosfamide (also known as TH302) as the hypoxia-activated prodrug. The obtained liposomes can help diagnose TNBC by fluorescence imaging and produce antitumor therapy by synergetic photodynamic therapy (PDT) and chemotherapy.

**Results:**

Compared with the nontargeted liposomes, the targeted liposomes exhibited good biocompatibility and targeting capability in vitro; in vivo, the targeted liposomes exhibited much better fluorescence imaging capability. Additionally, liposomes loaded with HPPH and TH302 showed significantly better antitumor effects than the other monotherapy groups both in vitro and in vivo.

**Conclusion:**

The impressive synergistic antitumor effects, together with the superior fluorescence imaging capability, good biocompatibility and minor side effects confers the liposomes with potential for future translational research in the diagnosis and CD44-overexpressing cancer therapy, especially TNBC.

**Graphic abstract:**

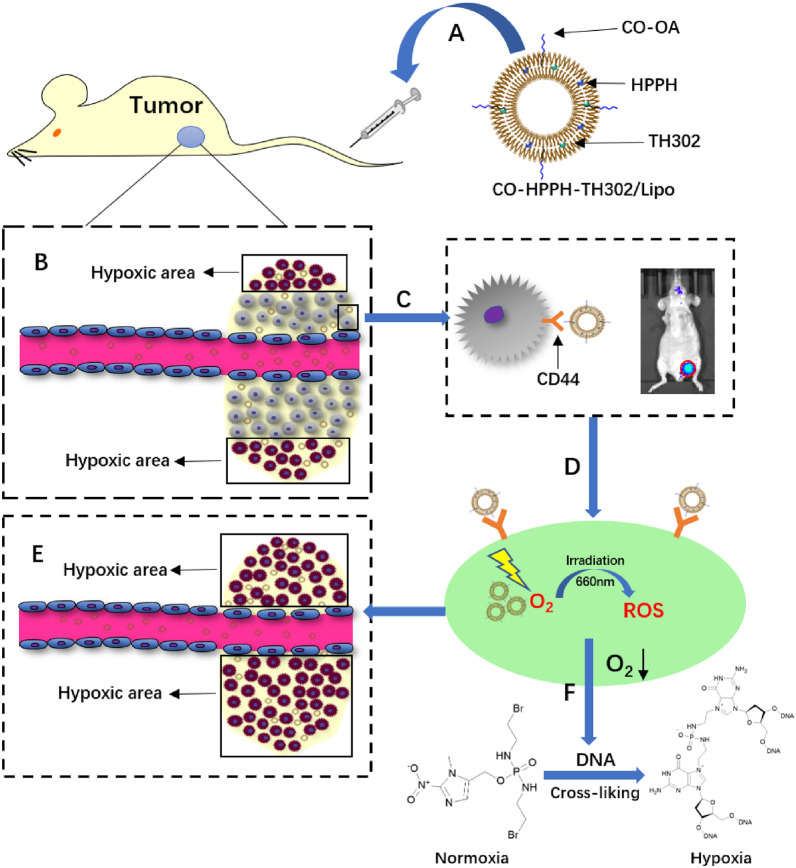

**Supplementary Information:**

The online version contains supplementary material available at 10.1186/s12951-021-00891-8.

## Background

Among all kinds of cancers, breast cancer is one of the biggest threats to women worldwide. Among its subtypes, triple-negative breast cancer (TNBC), which comprises 15–20% of the total instances of breast cancer, has on average a significantly worse prognosis than other subtypes owing to its potential invasiveness and risk of metastasis [1–7].

Traditional treatments fail due to the lack of estrogen receptors (ERs), progesterone receptors (PRs) and human epidermal growth factor receptors (HERs) [8, 9].

Hyaluronic acid receptor Cluster of differentiation-44 (CD44) is a kind of adhesion molecule that relates to tumorigenesis and tumor metastasis [10]. Zheng randomly sampled the clinical data of 139 cases of breast invasive ductal carcinoma (BIDC) and conducted a series of experiments, the results of which showed that TNBC expresses more CD44 than non-TNBC [11]. Therefore, many researchers chose CD44 as the therapeutic target of TNBC [12–17].

Chitosan oligosaccharide (CO), also known as hyaluronic acid analog, can specifically target breast cancer cells. In recent studies, some researchers have been devoted to CO-decorated nanoparticles targeting tumors overexpressing CD44, such as TNBC [17, 18].

Traditionally, treatments for TNBC include chemotherapy, radiotherapy, immunotherapy and surgery. The choice of treatment depends on the stage of the cancer. However, most treatments can cause a series of severe side effects, which are prone to cause pain or even death [19–26]. Photodynamic therapy (PDT) is a promising, novel, non-invasive medical technology that has been clinically proven to have dramatic effects with minimal side effects [27, 28]. PDT is based on the use of photosensitizers (PSs), and after being triggered by light of an appropriate wavelength, the PS can produce reactive oxygen species (ROS), which can induce cytotoxicity and eventually kill cancer cells. HPPH, as an effective PS, has several clinical advantages and shows promising efficacy and fluorescence imaging ability in preclinical studies [29–34]. Nevertheless, because of its inner mechanism, PDT is limited by the concentration of oxygen, which is also a major factor accounting for the failure in most treatments [35–37].

As one of the common pathological phenomena in the majority of solid tumors, hypoxia not only leads to a poor prognosis in many kinds of cancers but also limits the efficiency of traditional treatments [35, 37–40]. Evofosfamide (also known as TH302), a hypoxia-activated prodrug, has shown the ability to selectively kill cells by DNA damage in hypoxic tissues and significantly reduce the volume of tumors, especially tumors with massive hypoxic regions. Furthermore, TH302 can spread into peripheral normoxic regions once activated, which is called the bystander effect [41–45].

It is remarkable that PDT is not only an effective treatment for cancer but also provides a hypoxic environment for a hypoxia-activated prodrug [46]. In a study by Liu, they created a biodegradable liposome-based nanoparticle that combines PDT with the hypoxia-activated prodrug AQ4N for murine breast cancer (4T1) [47]. However, a new therapy for TNBC combining chitosan oligosaccharide-decorated liposomes targeting CD44^+^ cells with a sequential activation pattern via systemic administration remains a largely underexplored method.

**Scheme 1.**
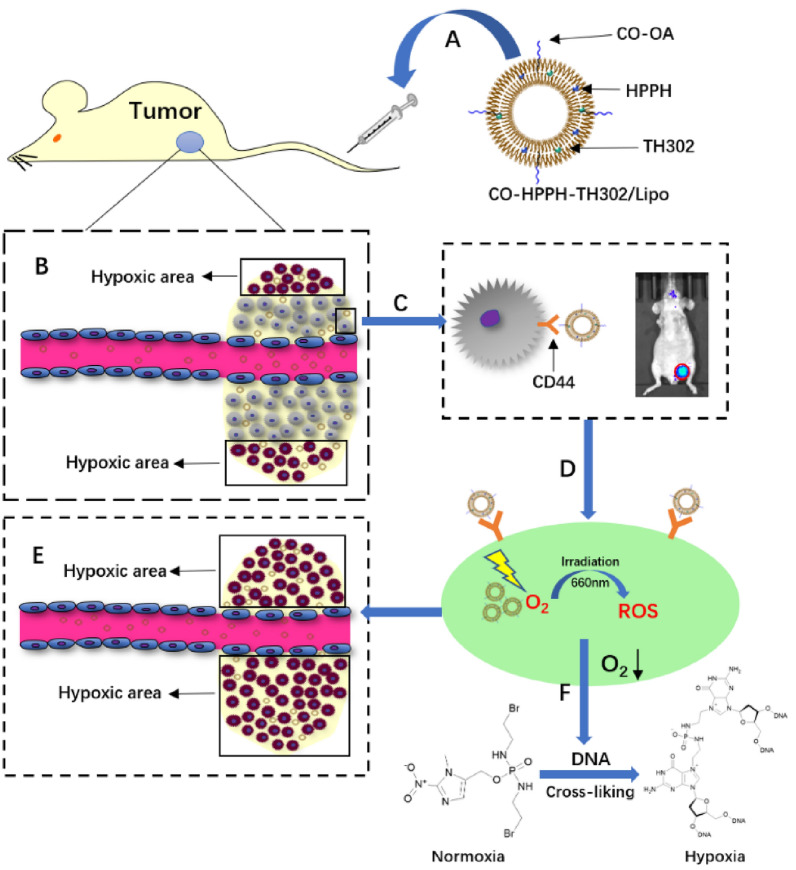
Schematic illustration of CO-HPPH-TH302/Lipo mediated synergetic chemotherapy/PDT in MDA-MB-231 tumor mouse model. The process includes five steps: **A** intravenous injection of CO-HPPH-TH302/Lipo via the tail vein; **B** liposomes accumulation in tumor tissue via passive targeting EPR effect; **C** active targeting via ligand–receptor-mediated endocytosis; **D** ROS generation to induce cell death via 660 nm irradiation; **E** transformation of normoxic area into hypoxic area after 660 nm irradiation; **F** DNA damage by activated TH302 at the hypoxic condition

In this paper, we developed a CO-modified nanoparticle based on liposomes by encapsulating the photosensitizer HPPH and the hypoxia-activated prodrug TH302 into hydrophobic bilayers (CO-HPPH-TH302/Lipo) (Scheme 1).

The results of the experiments proved that PDT and hypoxia-activated chemotherapy can be activated simultaneously by HPPH and TH302, and our CD44^+^-targeted liposomes effectively inhibited the growth of CD44-overexpressing tumors with minimal damage to normal tissues and helped to monitor tumors by fluorescence imaging. Such a CD44^+^ target therapeutic strategy is promising for future clinical applications.

## Results

### Preparation and characterization of CO-HPPH-TH302/Lipo

**Scheme 2.**
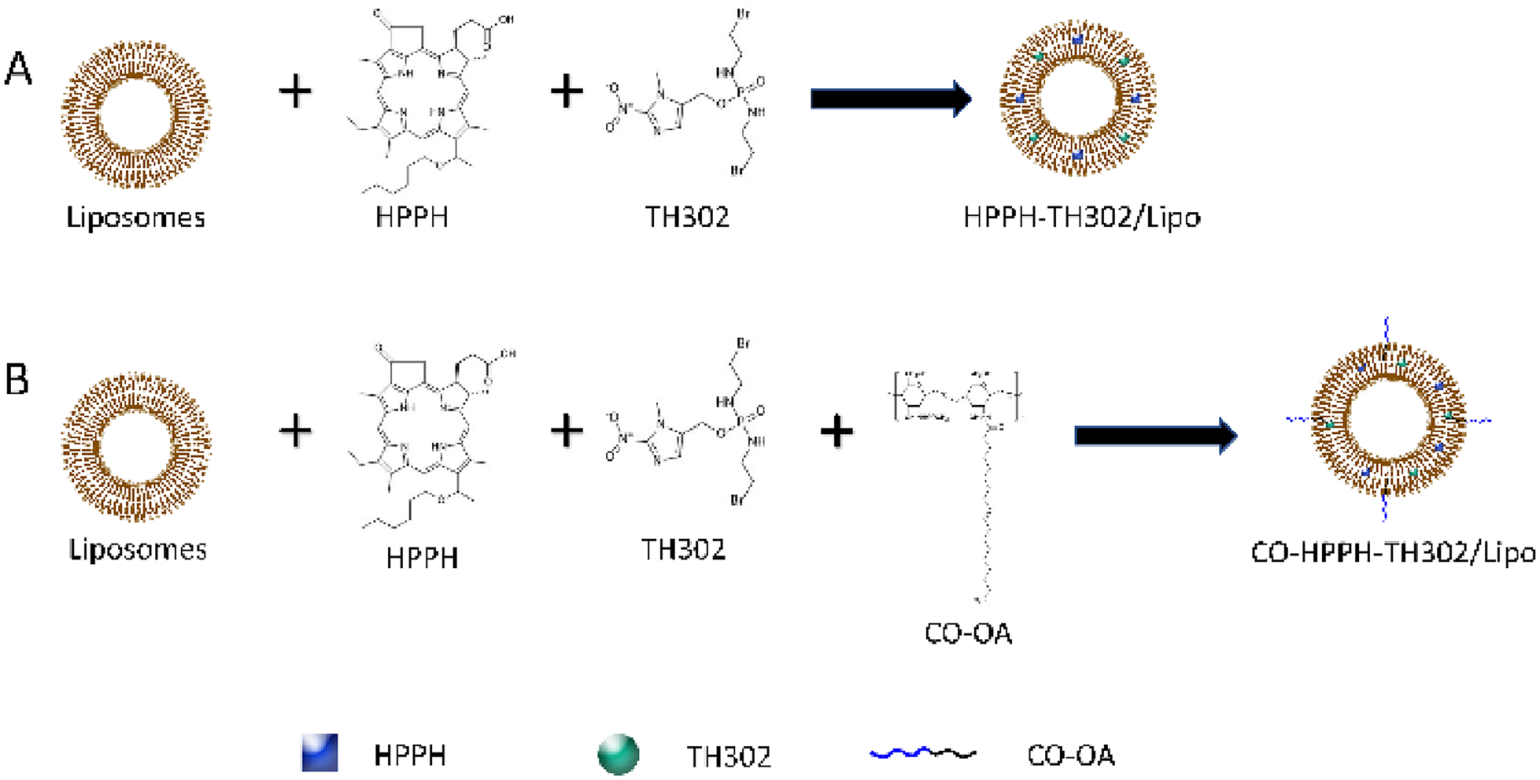
Schematic illustrations of the HPPH-TH302/Lipo (**A**) and CO-HPPH-TH302/Lipo (**B**) formation

**Fig. 1:**
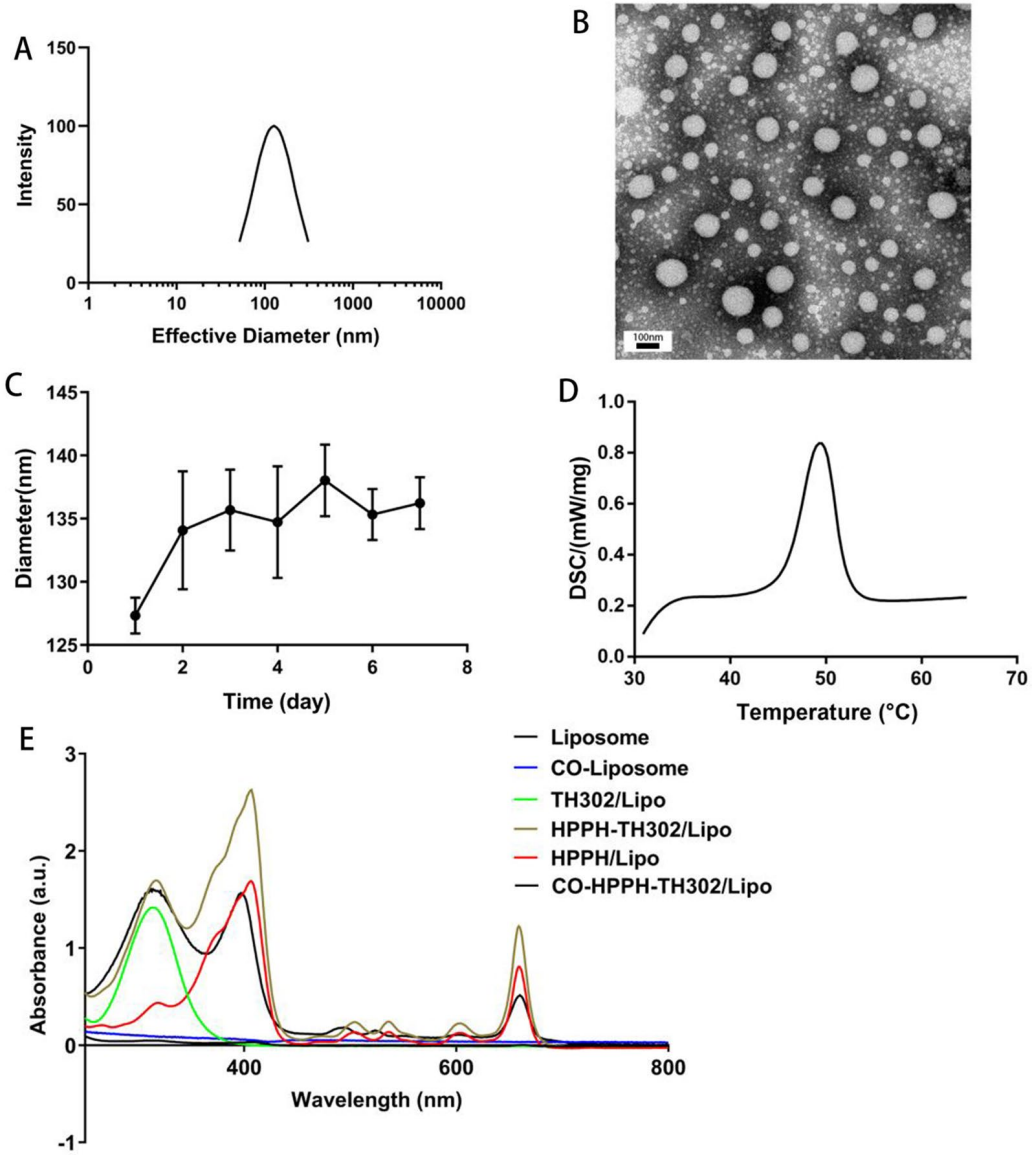
The characterization of HPPH-TH302/Lipo. **A** Size distributions of HPPH-TH302/Lipo in water. **B** Surface morphology of HPPH-TH302/Lipo in TEM. **C** DLS size measurements of HPPH-TH302/Lipo in PBS for 7 days, each bar represents the mean ± SD of three replicates. **D** The phase transition temperature of HPPH-TH302/Lipo. **E** Spectrophotometer results of Liposome, CO-Liposome, TH302/Lipo, HPPH-TH302/Lipo, HPPH/Lipo and CO-HPPH-TH302/Lipo

**Fig. 2:**
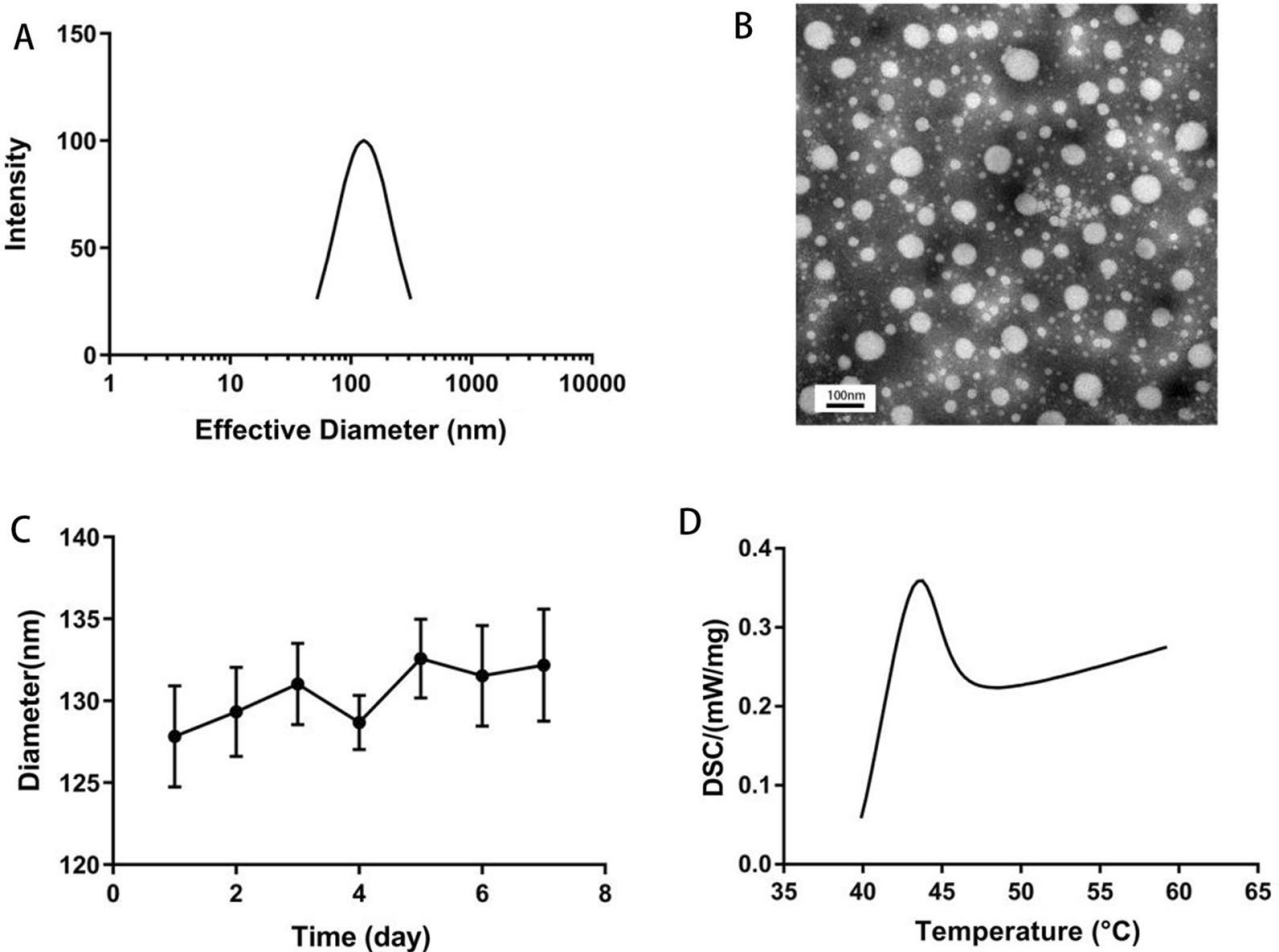
The characterization of HPPH-TH302/Lipo. **A** Size distributions of CO-HPPH-TH302/Lipo in water. **B** Surface morphology of CO-HPPH-TH302/Lipo in TEM. **C** DLS size measurements of CO-HPPH-TH302/Lipo in PBS for 7 days, each bar represents the mean ± SD of three replicates. **D** The phase transition temperature of CO-HPPH-TH302/Lipo

We prepared the water-soluble CO-OA coated photosensitive liposome CO-HPPH-TH302/Lipo (Scheme 2). OA was inserted into the surface of liposomes by the theory of “similarity and intermiscibility”, and CO was exposed outside of the liposome. According to the thin-layer chromatography (TLC) experiment in Additional file 1: Figure S1A, OA was completely consumed and became OA-NHS with smaller polarity. In Additional file 1: Figure S1B, OA-NHS was reacted completely to give the CO-OA as the reaction product. As the results shown of HPLC–MS analysis in Additional file 1: Figure S2, the purity of HPPH lipid was 98.126%. HPPH and TH302 were encapsulated in the phospholipid bilayer because of the lipid-soluble feature. As shown in Figs. 1A and 2A, the diameters of HPPH-TH302/Lipo and CO-HPPH-TH302/Lipo in water were 127.8 ± 75.1 nm and 128.7 ± 75.0 nm, respectively. According to the TEM images shown in Figs. 1B and 2B, the morphologies of HPPH-TH302/Lipo and CO-HPPH-TH302/Lipo were spherical, and the average size was approximately 80–150 nm, which matched the particle size described previously. The stability of HPPH-TH302/Lipo and CO-HPPH-TH302/Lipo were monitored (Figs. 1C, 2C), and the particle size changed very slightly over 7 days, which suggests that both particles have good stability. To examine the encapsulation of the different compositions in various liposomes, UV–Vis spectrometry was used (Fig. 1E**)**. HPPH-TH302/Lipo and CO-HPPH-TH302/Lipo both had characteristic peaks at 331 nm and 660 nm, which were the characteristic peaks for TH302 and HPPH, suggesting that the drugs were successfully loaded; the encapsulation rates of TH302 and HPPH were 82% and 50%, respectively. However, we still cannot confirm whether the liposome linked CO directly. To further verify that CO was successfully linked to the surface of the liposome, HPPH-TH302/Lipo and CO-HPPH-TH302/Lipo were examined by differential scanning calorimetry (DSC), and the phase transition temperatures of HPPH-TH302/Lipo and CO-HPPH-TH302/Lipo were 49.3 °C and 43.5 °C, respectively (Figs. 1D, 2D). Because of the different compositions of the phospholipid bilayer, the phase transition temperature changed significantly, which could indirectly prove that CO was linked to the surface of the liposome. What’s more, we further measured the zeta potentials of HPPH-TH302/Lipo and CO-HPPH-TH302/Lipo. The zeta potentials of HPPH-TH302/Lipo and CO-HPPH-TH302/Lipo were − 10.16 ± 3.38 mV and 29.97 ± 3.5 mV, respectively. The apparently charge change between HPPH-TH302/Lipo and CO-HPPH-TH302/Lipo demonstrated the successful synthesis of CO-HPPH-TH302/Lipo (Additional file 1: Figure S3).

### Singlet oxygen generation ability in vitro

**Fig. 3:**
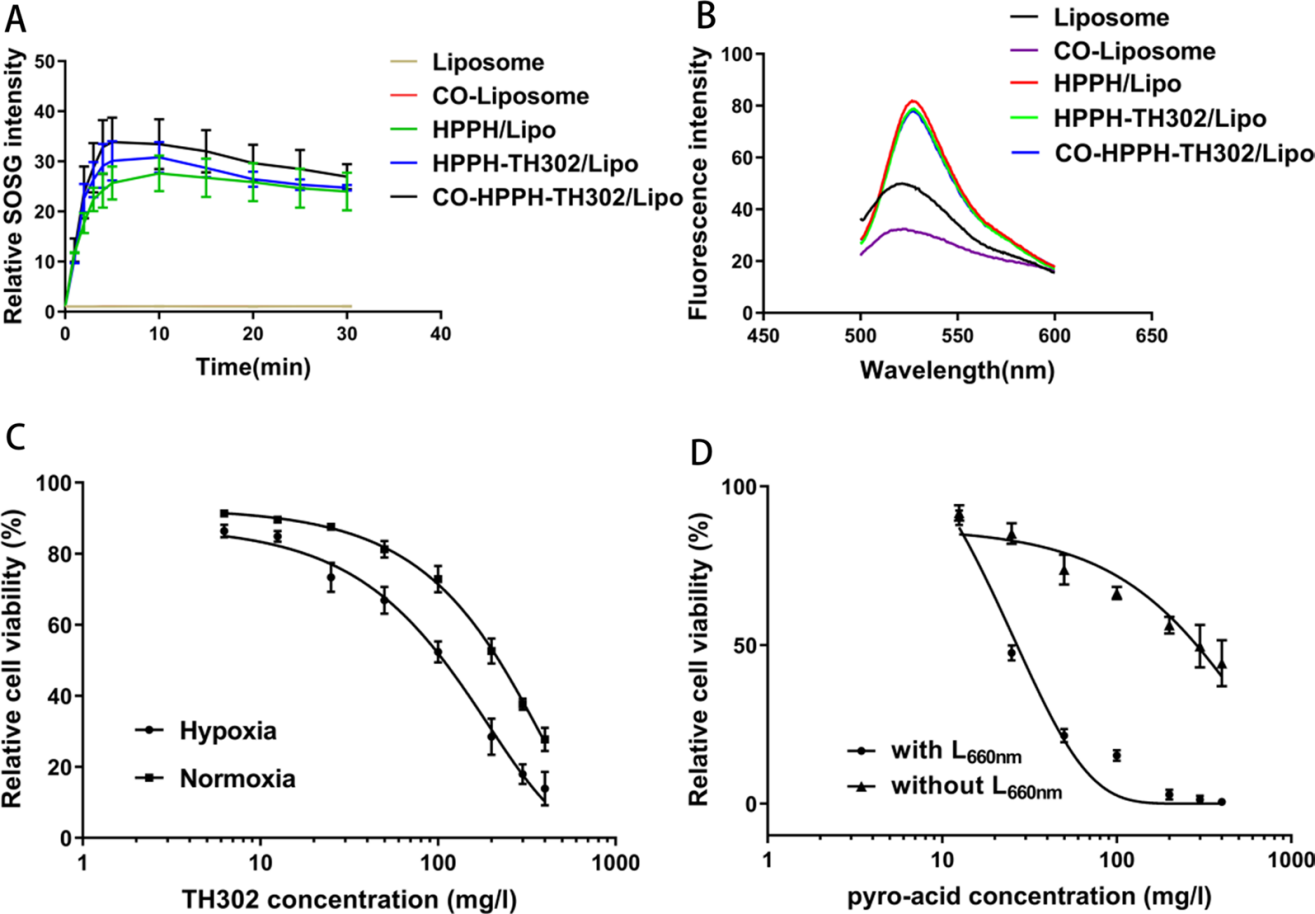
The IC50 of TH302 and HPPH and singlet oxygen generation ability of various liposomes in vitro. **A** Singlet oxygen generation abilities of Liposome, CO-Liposome, HPPH/Lipo, HPPH-TH302/Lipo and CO-HPPH-TH302/Lipo determined by using SOSG; **B** fluorescence emission of various liposomes determined by DCFH-DA probe, each bar represents the mean ± SD of three replicates. **C** The half-inhibitory concentration of TH302 with and without oxygen; **D** the half-inhibitory concentration of HPPH with and without irradiation, each bar represents the mean ± SD of three replicates

Photodynamic therapy causes damage of tumor cells by producing tremendous cytotoxic ROS [48]. To confirm that the various liposomes with HPPH can generate singlet oxygen and to determine the optimal exposure time, the SOSG assay was performed in various liposomes in vitro. As shown in Fig. 3A, the relative SOSG intensity increased rapidly after irradiation, and the optimal exposure time was 10 min, which suggests that 10 min is a reasonable exposure time for the following experiments in vitro. In addition, we detected the electron spin resonance (ESR) spectrum by electron paramagnetic resonance spectrometer, which is an authoritative characterization of ROS [49, 50]. Although all kinds of liposomes showed nearly no signals before irradiation, the typical triplet signal emerged in only the groups containing HPPH after irradiation, which directly comfirmed the singlet oxygen can be produced by CO-HPPH-TH302/Lopo after irradiation. (Additional file 1: Figures S4, S5). What’s more, according to the results of ROS Assay Kit, the liposomes loaded with HPPH can generate singlet oxygen, which also suggests that the drug was loaded successfully (Fig. 3B).

### Half-inhibitory concentration (IC50) in vitro

In order to determine the optimal drug concentration in vitro, the IC50 values of TH302 with and without oxygen were 0.2 mg/mL and 0.1 mg/mL, respectively (Fig. 3C). The IC50 values of HPPH with and without laser irradiation were 0.025 mg/mL and 0.3 mg/mL, respectively (Fig. 3D). According to the study above, the most suitable concentrations of TH302 and HPPH were 0.1 mg/mL and 0.025 mg/mL for the following in vitro experiments.

### Cellular uptake in vitro

**Fig. 4:**
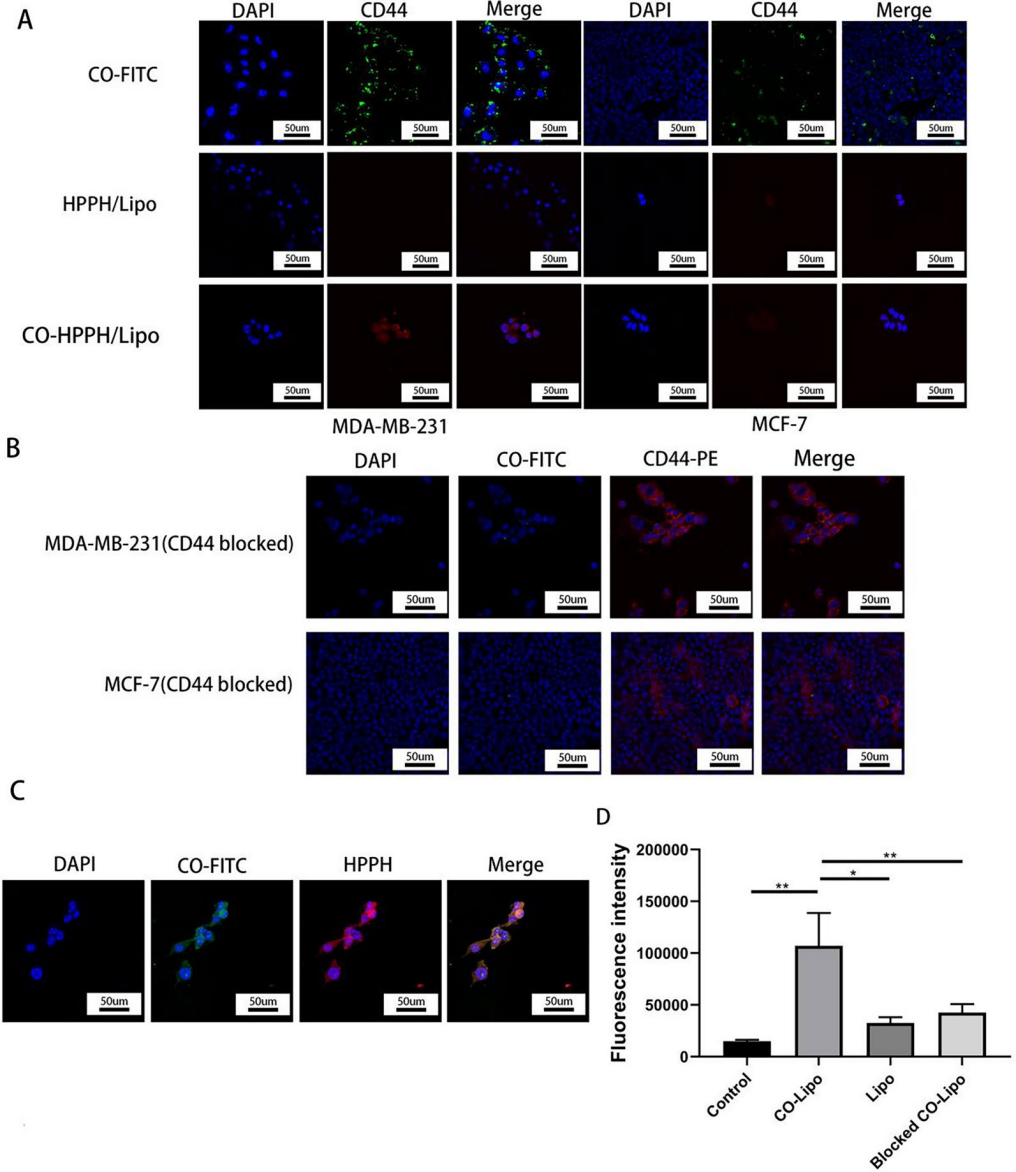
The results of targeting ability of the CO coated liposomes in vitro. **A** MDA-MB-231 and MCF-7 cells were treated with CO-FITC, HPPH/Lipo (i.e., nontargeted group) and CO-HPPH/Lipo (i.e., targeted group). Upon 400 nm irradiation, the HPPH emits red luminescence from the cytoplasm of the cells treated with HPPH/Lipo and CO-HPPH/Lipo. **B** MDA-MB-231 and MCF-7 cells were blocked by anti-CD44-PE, then treated with CO-FITC respectively. Upon 480 nm irradiation, the PE emits red luminescence from the cell membrane of the cells treated with anti-CD44-PE. **C** MDA-MB-231 cells were treated with FITC-CO-TH302-HPPH/Lipo. Upon 400 nm irradiation, the HPPH emits red luminescence from the cytoplasm of the cells treated with CO-HPPH/Lipo. Blue luminescence is from the nucleus after being stained with DAPI. Upon 495 nm irradiation, the FITC emits green luminescence from the cell membrane of the cells treated with CO-FITC (**A**) and FITC-CO-HPPH/Lipo (**C**). Scale bar: 50 µm. **D** Mean fluorescence intensities (MFIs) in different groups according to the flow cytometric results, each bar represents the mean ± SD of five replicates. * means P < 0.05, ** means P < 0.01, and *** means P < 0.001

As far as we know, the targeting ability of nanoparticles is very critical for biomedical applications. To examine the binding ability of CO-HPPH-TH302/Lipo with CD44-overexpressing tumors in vitro, the TNBC cell line MDA-MB-231 was used as the experimental group, and the non-TNBC cell lines MCF-7, CD44-blocked MCF-7 cells and CD44-blocked MDA-MB-231 cells were used as the control groups with lower CD44 expression than the experimental group. The CO-FITC group and CO-HPPH/Lipo group showed much greater cellular uptake ability in the MDA-MB-231 TNBC cell line than that in the MCF-7 non-TNBC cell line, and the groups without CO showed very little uptake in both cell lines with the same incubation time (Fig. 4A). It was clear from the data shown above that liposomes coated with CO could increase the cellular uptake ability of liposomes compared with liposomes without CO. As shown in Fig. 4B, CO barely appeared at the surface of cells that were blocked by the anti-CD44 antibody. The results further verified that CO could effectively target CD44. In addition, Fig. 4C demonstrates that CO-HPPH-TH302/Lipo could effectively target TNBC and further verified that CO was successfully linked to the liposome. In order to further assess the binding ability between targeted and non-targeted liposomes, we have conducted flow cytometry and measured the fluorescence intensity. The MFIs of CO-Lipo group (107,012.33 ± 31,762.13) was about three times that of Lipo group (32,513.33 ± 5569.32) and Blocked CO-Lipo group (42,640 ± 8039.54), respectively (Fig. 4D, Additional file 1: Figure S6). The results of the cellular uptake experiments showed that CO was successfully linked to the liposome and could target CD44-overexpressing cells.

### Fluorescence imaging in vivo

**Fig. 5:**
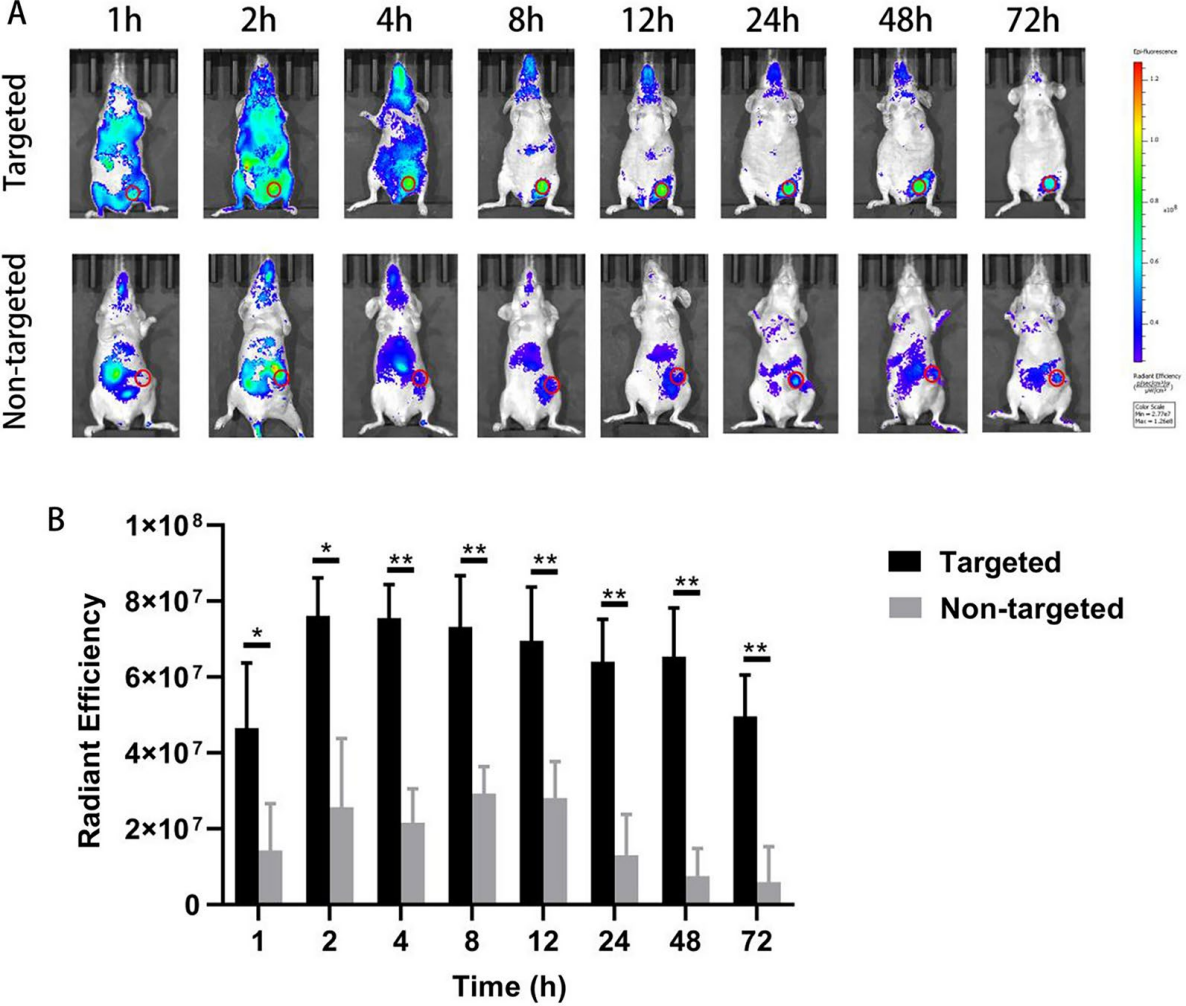
The results of targeting ability of the CO coated liposomes in vivo. **A** Distribution of CO-HPPH-TH302/Lipo (i.e., targeted group) and HPPH-TH302/Lipo (i.e., nontargeted group) in tumor-bearing mice. The small red circles are the tumor regions(ROI). **B** Comparison of the radiant efficiencies between targeted and nontargeted groups at the different time points, each bar represents the mean ± SD of three replicates. * means P < 0.05, ** means P < 0.01, and *** means P < 0.001

We had verified that CO coated liposomes had significant CD44-overexpressing cell targeting ability in vitro, and the targeting ability depended on the binding of CO with CD44. To further evaluate the targeting ability of CO coated liposomes in vivo, CO-HPPH-TH302/Lipo and HPPH-TH302/Lipo were injected into MDA-MB-231 cell tumor-bearing mice through the tail vein. The intensity of fluorescence and the distribution of two types of liposomes were recorded at different time points (1 h, 2 h, 4 h, 8 h, 12 h, 24 h, 48 h, 72 h) in vivo. Then the radiant efficiencies of tumor regions were measured. As shown in Fig. 5A, the fluorescence signal was strongly accumulated in the liver and spleen 2 h post-injection, the signal of CO-HPPH-TH302/Lipo group was gradually decreased 8 h post-injection, disappeared 24 h post-injection. Moreover, the fluorescence signal of HPPH-TH302/Lipo group was also strongly accumulated in the liver and spleen 2 h post-injection and gradually decreased at the following time points, there was still fluorescence signal until 72 h after injection. What’s more, the fluorescence signal of tumor area was much stronger in the CO-HPPH-TH302/Lipo group than in the HPPH-TH302/Lipo group 4 h post-injection, which indicated that CO-HPPH-TH302/Lipo possessed active targeting ability. Compared to the fluorescence signals at the next 5 time points in the CO-HPPH-TH302/Lipo group and HPPH-TH302/Lipo group, fluorescence signals of the HPPH-TH302/Lipo group were much weaker in the tumor areas than CO-HPPH-TH302/Lipo group, demonstrated that HPPH-TH302/Lipo group only exhibited the passive targeting ability via the enhanced permeability and retention effect (EPR) effect, and the CO-HPPH-TH302/Lipo possessed both active targeting ability and the EPR effect, leading to high liposome tumor accumulation even after 72 h. As shown in Fig. 5B, the radiant efficiency in the tumor area of the CO-HPPH-TH302/Lipo group enhanced rapidly, and reached the peak at approximately 2 h post-injected, whereas there was still a large amount of fluorescence until 48 h. Similarly, the fluorescence signal in the tumor area of the CO-HPPH-TH302/Lipo group also enhanced rapidly, however, the signal degraded rapidly after 12 h, which was much earlier than the CO-HPPH-TH302/Lipo group. Taken together, this study has demonstrated that the CO-HPPH-TH302/Lipo can effectively target CD44-overexpressing cells (e.g. TNBC), which is promising for the future study.

### Cytotoxicity in vitro

**Fig. 6:**
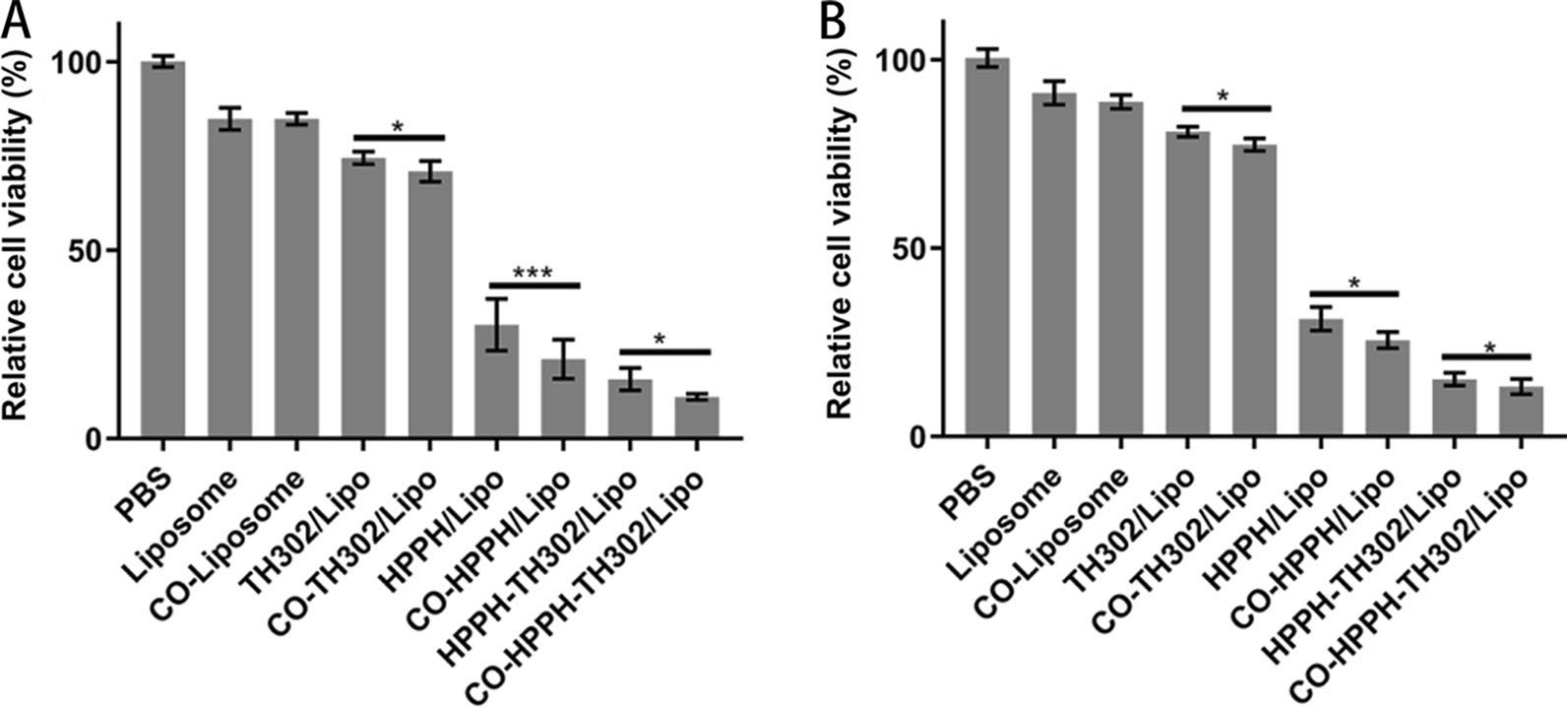
The cytotoxicity of various liposomes in vitro. **A** Inhibition of viability of MDA-MB-231 cells; **B** inhibition of viability of MCF-7 cells, each bar represents the mean ± SD of five replicates. * means P < 0.05, ** means P < 0.01, and *** means P < 0.001

The cytotoxicity of various liposomes toward MDA-MB-231 and MCF-7 cells was evaluated by the CCK-8 assay. Figure 6 showed that the relative MDA-MB-231 cell viability rates of the liposome group and CO-liposome group were 84.85 ± 2.95% and 84.83 ± 1.53%, respectively. The relative MCF-7 cell viability rates of the liposome group and CO-liposome group were 91.18 ± 3.11% and 88.79 ± 1.87%, respectively. All these data indicated that the liposomes possessed good biocompatibility. The MDA-MB-231 cell viability rates of the TH302/Lipo group and CO-TH302/Lipo group were 74.48 ± 1.63% and 70.87 ± 2.74%, respectively. The MCF-7 cell viability rates of the TH302/Lipo group and CO-TH302/Lipo group were 80.85 ± 1.34% and 77.43 ± 1.67%, respectively. After TH302 loading, the toxicity of the TH302/Lipo group and CO-TH302/Lipo group in MDA-MB-231 and MCF-7 cells increased slightly because it was difficult to create a hypoxic environment to activate TH302 in vitro, and because of the low CD44 expression, there was no significant difference between the TH302/Lipo group and CO-TH302/Lipo group in MCF-7 cells. The MDA-MB-231 cell viability rates of the HPPH/Lipo group and CO-HPPH/Lipo group were 30.20 ± 6.87% and 21.09 ± 5.16%, respectively. The MDA-MB-231 cell viability rates of the HPPH-TH302/Lipo group and CO-HPPH-TH302/Lipo group were 15.75 ± 2.99% and 11.08 ± 0.79%, respectively. The MCF-7 viability rates of the HPPH/Lipo group and CO-HPPH/Lipo group were 31.20 ± 3.11% and 25.57 ± 2.14%, respectively. The MCF-7 viability rates of the HPPH-TH302/Lipo group and CO-HPPH-TH302/Lipo group were 15.21 ± 1.66% and 13.26 ± 2.00%, respectively. The cell viability rate decreased significantly after PDT, and the cytotoxicity of the HPPH-TH302/Lipo group and CO-HPPH-TH302/Lipo group significantly increased because PDT consumed oxygen and created a hypoxic environment to activate the hypoxia-activated prodrug TH302, further killing tumor cells. In addition, comparing the cytotoxic ability of the target PDT group and nontarget PDT group in MDA-MB-231 cells, the cell viability rate of the target PDT groups was much lower than that of the nontarget PDT groups in MDA-MB-231 cells, and because of the low CD44 expression, the cytotoxicity of the target PDT groups and nontarget PDT groups showed no significant difference in MCF-7 cells. Comparing the cytotoxicity of the HPPH-TH302/Lipo group and CO-HPPH-TH302/Lipo group in MDA-MB-231 cells, the target group reflected a better antitumor effect than the nontarget group; however, because of the low CD44 expression, there was a minor difference in the effects of the target groups and nontarget groups in MCF-7 cells, and the results indicated that PDT and TH302 synergistic therapy has extreme destruction efficiency in vitro. In addition, Additional file 1: Figure S7 showed that after the illumination, there was no significant cytotoxicity in the groups without HPPH, which also demonstrated that 660 nm light has no obvious phototoxicity in vitro. What’s more, we dilute various liposomes with lower concentration. As shown in Additional file 1: Figure S8, the group which is diluted to half also shows that the effect of combination therapies is better than that of single-drug ones. However, due to the low drug concentration, the therapeutic effect is limited, and there are no significant differences from the other two groups with lower concentrations.

### Antitumor effects in vivo

**Fig. 7:**
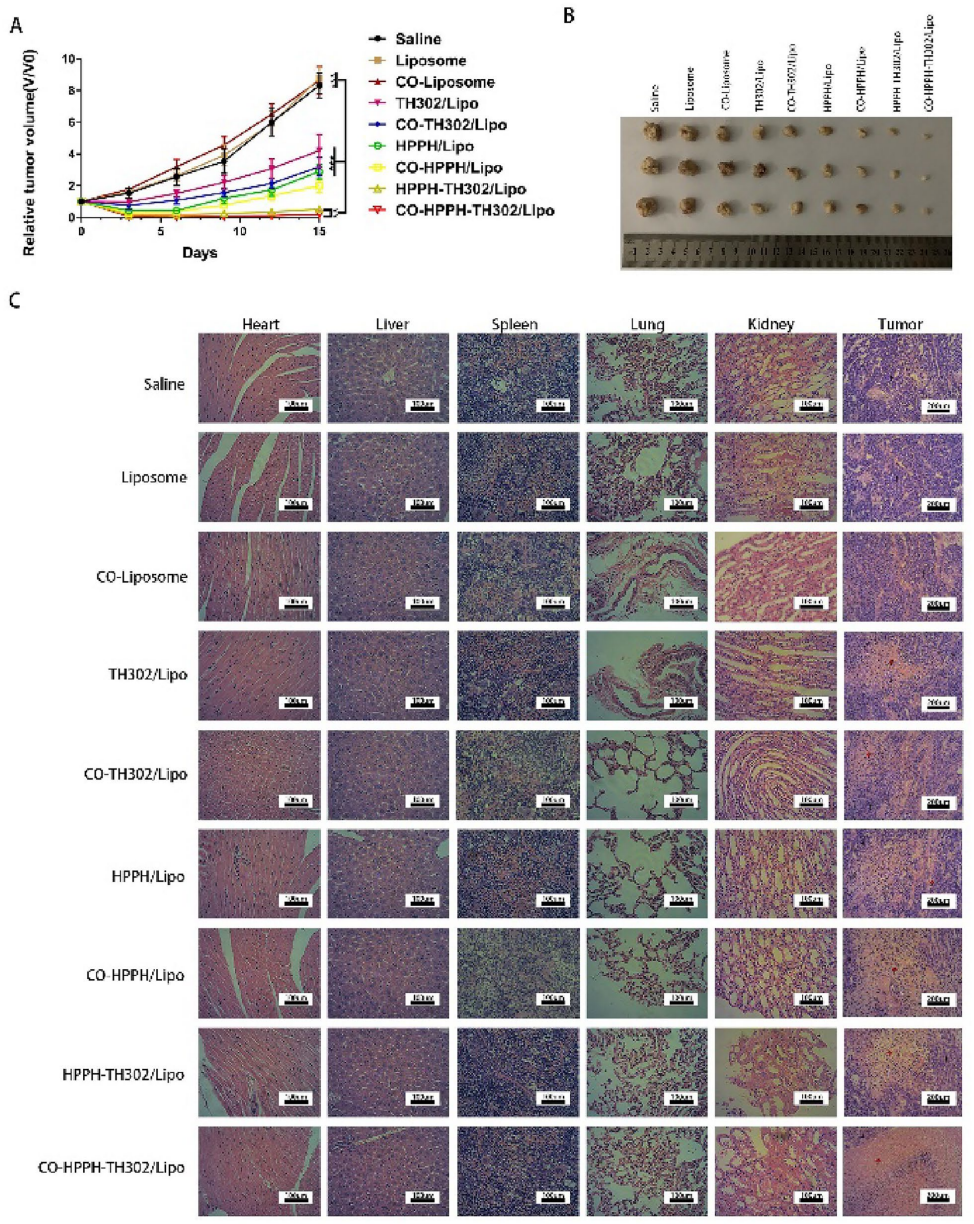
Antitumor effects in vivo, **A** tumor growth curves of mice after various different treatments as indicated. V and V_0_ stand for the tumor volumes after and before the treatment, each bar represents the mean ± SD of six replicates. * means P < 0.05, ** means P < 0.01, and *** means P < 0.001. **B** Photographs of tumors collected from different groups, 15 days after the treatment. **C** Tumor tissues of MDA-MB-231-bearing mice stained by H&E (the black arrows indicate the tumor tissues; the red arrows indicate the necrosis and collapse of tumor cells). Scale bars = 200 µm. The main organs of MDA-MB-231-bearing mice treated by various experimental groups stained by H&E. Scale bars = 100 µm

The effects of CO-HPPH-TH302/Lipo on MDA-MB-231 tumor growth was further evaluated by calculating the changes in tumor volume and by analysis of the relative tumor volume. The tumors in mice that were treated with CO-HPPH-TH302/Lipo showed the best therapeutic effect among all the treatment groups. In addition, the relative tumor volume was not obviously different among the saline group, liposome group and CO-liposome group, which indicates that both liposomes and CO are harmless. We further compared the monotherapy groups with the combined treatment groups. Tumor growth was significantly limited in the combined treatment groups, and the monotherapy groups showed poorer therapeutic effects than the combined treatment groups. Moreover, the target groups displayed greater tumor suppressive effects than the nontarget groups (Fig. 7A, B). Moreover, based on the images of hematoxylin and eosin (H&E) staining, the group treated with CO-HPPH-TH302/Lipo demonstrated the most remarkable necrosis and severe morphology change (Fig. 7C), which also further proves the effect of CO-HPPH-TH302/Lipo group is much better than the other groups.

To further confirmed that the liposomes and the therapeutic strategy possessed the advantages of biological safety. After the treatment, there was no obvious skin damage, which means 660 nm light illumination barely have the phototoxicity in vivo. Furthermore, we also observed the body weight of tumor-bearing nude mice in each group after treatment. As shown in Additional file 1: Figure S9, there were no significant changes in the groups with PDT. HE staining results (Fig. 7C) showed that compared with the saline group, the heart, liver, spleen, lung and kidney in various experimental groups exhibited no obvious tissue damage, which verifies the biological safety of the various groups. Two reasons cause this phenomenon that there is no difference between the target groups and nontarget groups: (1) PDT needs to be activated by the proper laser; and (2) normal tissues with a large content of oxygen cannot activate the hypoxia-activated prodrug. In summary, CO-coated liposomes and the therapeutic strategy of exogenous stimulation significantly reduce the side effects and ensure the safety of liposomes.

## Discussion

Clinically, endocrine therapy is the best treatment for most types of breast cancer. However, triple-negative breast cancer is not sensitive to endocrine therapy due to its special pathological features [51]. Over the past decades, chemotherapy has been the main treatment for triple-negative breast cancer, which causes several serious side effects [52]. In order to solve the problem mentioned above, many papers have adopted various methods to reduce the side effects of chemotherapy [53]. In our research, we used the following two methods to improve the specificity and targeting ability to reduce the side effects of treatment: (1) we modified CO onto the surface of nanomaterials to actively target the high expression of CD44 in triple-negative breast cancer, and (2) through photodynamic therapy, we used 660 nm light to actively irradiate the tumor site.

Photodynamic therapy has been approved by the Food and Drug Administration (FDA) and in many other countries as a clinical therapy, but the therapeutic effects of photodynamic therapy are often limited by hypoxia [54]. There are several treatment strategies to solve the problem of hypoxia in photodynamic therapy: (1) actively increase the amount of oxygen in the tumor tissue area; (2) reduce oxygen consumption in the tumor area; or (3) activate hypoxia-activated prodrugs in the hypoxic region. In our study, TH302, a hypoxia-activated prodrug, took advantage of the hypoxic environment produced by photodynamic therapy for activation. In addition, we used the fluorescence imaging of photosensitizers to observe the therapeutic effects on tumor tissue.

Compared with other nanoparticles, liposomes have several advantages: (1) good biocompatibility, (2) easy preparation, (3) excellent drug loading capacity, which can contain both water-soluble and lipid-soluble drugs, and (4) drug delivery targeting through surface modification [55, 56]. At present, many liposome-based drugs, such as pegylated liposomal doxorubicin, have been used as antitumor drugs in the clinic [57]. Based on the advantages above, we prepared the photosensitive liposome CO-HPPH-TH302/Lipo. Due to the lack of targeting ability, conventional unmodified liposomes are not able to efficiently deliver drugs to the tumor tissues, which will cause side effects. Therefore, through the surface modification of liposomes, the side effects of the drugs can be reduced by targeting triple-negative breast cancer through CD44. It has been reported that chitosan oligosaccharide-modified nanomaterials can specifically target breast cancer stem cells and solve the problem of recurrence of breast cancer after conventional chemotherapy [18]. Therefore, we modified chitosan oligosaccharide on the surface of liposomes to target CD44-positive tumor cells.

In this study, we first characterized the nanoparticles, including their particle size, zeta potential, morphology, stability and phase transition temperature, in each group and evaluated the drug loading capacity of the liposomes. As an excellent nanocarrier, the liposomes showed good drug loading capacity and stability.

After that, we verified the targeting ability of CO-HPPH-TH302/Lipo. In vitro experiments, we prepared both targeted liposomes and nontargeted liposomes. The results showed that targeted liposomes had a better targeting ability for triple-negative breast cancer highly expressing CD44. In order to further confirm the targeting ability of CO-HPPH-TH302/Lipo, we used fluorescence imaging to verify the targeting ability of the targeted liposomes and nontargeted liposomes in triple-negative breast cancer-bearing mice. The fluorescence intensity of the tumor area in the targeted group was much higher than that in the nontargeted group. In conclusion, CO-HPPH-TH302/Lipo can specifically target cancer cells highly expressing CD44, increase the drug concentration in tumor tissues and reduce drug aggregation in nontarget tissues.

Based on the results shown above, we further explored the therapeutic effects of CO-HPPH-TH302/Lipo in vitro and in vivo. A cell counting kit-8 (CCK-8) assay was used to evaluate the therapeutic effects of the different liposomes in vitro, which revealed that photodynamic therapy combined with a hypoxia-activating drug had the best therapeutic effects. Moreover, the in vivo therapeutic effects of the targeted combination therapy group were better than that of the other groups.

CO-HPPH-TH302/Lipo showed good targeting ability and therapeutic effects in triple-negative breast cancer. In order to achieve clinical translation, we need to observe and optimize the dosage, treatment time and treatment cycle and evaluate the 660 nm light penetration ability for photodynamic therapy. In addition, it is necessary to further evaluate the long-term efficacy of treatment and the therapeutic effects in large animals and clinical trials. Clinically, the low penetration of lights limits the therapeutic depth of PDT. With the help of optical fibers, endoscopes and interventional techniques, laser can be guided to the deep part of the body for treatment, therefore avoiding the traumas and pains caused by thoracotomy, laparotomy and other operations. Recently, a wireless implantable photonic device has been reported for therapeutic light delivery in PDT, which provides a good idea to solve the limitations of photodynamic therapy [58]. For breast cancer, we can use ultrasound-guided percutaneous puncture to insert multiple optical fibers around the tumor tissue for PDT, so as to reduce the attenuation of illumination intensity. What’s more, given the heterogeneity of tumors, it is not feasible to guarantee the effect of photodynamic therapy on tumors of different depths. In order to deliver better clinical applications, we need to provide personalized treatments for different patients. To begin with, it is possible to use imaging techniques to determine the location and size of a tumor. Afterwards, a treatment plan can be made to determine the illumination dose. Then, guided by ultrasound, we insert multiple optical fibers around the tumor tissue for photodynamic therapy. Finally, the follow-up treatments shall be adjusted according to the previous treatment effect. Nowadays, many photosensitizers have been clinically approved for cancer therapy, and many liposomes have been approved by FDA as well [54, 59–61]. Therefore, CO-HPPH-TH302/Lipo is promising for translation into the clinical practice of TNBC treatment.

## Conclusion

In this study, we successfully synthesized the multifunctional compound CO-HPPH-TH302/Lipo, which efficiently combines CD44 targeting, fluorescence imaging, photodynamic therapy and hypoxia-activated prodrug synergistic therapy. This synergistic therapy managed to amplify the effects of each individual therapy as well as reduce the side effects in vitro and in vivo. The novel nanoparticle possesses good biocompatibility and excellent destruction ability.

In summary, CO-HPPH-TH302/Lipo can be used as a potentially precise diagnostic and therapeutic vector for CD44-overexpressing tumors, such as TNBC, with an extremely effective therapeutic strategy.

## Materials

### Chemicals and agents

1-Palmitoyl-2-hydroxy sn-glycero-3-phosphocholine (P-lyso-PC, LPC) was purchased from Corden Pharma (Switzerland). Cholesterol was purchased from Shanghai Huixing Biochemical Reagents Co., Ltd. (Shanghai, China). TH302 was purchased from Efebio Co., Ltd. (Shanghai, China). HPPH was purchased from MCE (MedChemExpress LLC, USA). Oleic acid (OA), *N*-(3-(dimethylamino)-propyl)-*N*-ethylcarbodiimide hydrochloride crystalline (EDC), *N*-hydroxysuccinimide (NHS), *N*,*N*-diisopropylethylamine (DIPEA) and 4-(dimethylamino) pyridine (DMAP) were purchased from Sigma-Aldrich (St. Louis, USA). Chitosan oligosaccharide (CO) was purchased from TCI (Shanghai, China). Chloroform, acetic acid and methanol were purchased from Sinopharm Chemical Reagent Co., Ltd. (Shanghai, China). CO-FITC and FITC-CO-HPPH-TH302/Lipo were purchased from Xi’an Ruixi Biological Technology Co., Ltd. (Xi’an, China). Anti-CD44-PE and singlet oxygen sensor green (SOSG) were purchased from Invitrogen, Thermo Fisher Scientific (Waltham, MA, USA). A reactive oxygen species assay kit 2′-7′dichlorofluorescin diacetate (DCFH-DA probe) was purchased from Beyotime Biotechnology (China). All solvents were of high-performance liquid chromatography grade.

### Cell culture

The MDA-MB-231 and MCF-7 cell lines were purchased from the Cell Bank of the Chinese Academy of Sciences (Shanghai, China). MDA-MB-231 and MCF-7 cells were cultured in RPMI 1640 (Gibco, Grand Island, NY, USA), supplemented with 10% fetal bovine serum (FBS, Thermo Fisher Scientific), 100 U/mL penicillin, and 100 mg/mL streptomycin (Thermo Fisher Scientific), and were maintained at 37 °C with 5% CO_2_ in a humidified incubator.

### Synthesis of the HPPH lipid

The HPPH lipid was prepared as described by Zheng [33]. First, 100 nmol of LPC, 50 nmol of HPPH, 50 nmol of EDC, 25 nmol of DAMP and 50 µL of DIPA were combined in 10 mL of anhydrous dichloromethane. The reaction mixture stirred at room temperature under argon in the dark for 48 h. Purification was achieved by using diol-modified silica (Sorbtech) and eluting the product with 8% methanol in DCM after washing out impurities with 2% and 5% methanol in DCM. The HPPH lipid was then dried under nitrogen and stored under argon at − 20 °C in 1 µmol aliquots. The purity of HPPH lipid was analyzed by HPLC–MS/MS (LCMS-8030, Shimadzu, Japan) equipped with a C8 column. The mobile phase solutions consisted of (A) methanol and 0.1% formic acid and (B) water and 0.1% formic acid. Elution was with 0.8 mL/min flow, starting at 40% A, ramping to 100% A over 10 min and continued with 100% A for 5 min. Detection wavelength was 420 nm.

### Synthesis of the oleic acid-conjugated chitosan oligosaccharide

OA-CO was prepared as described by Zhang [62, 63]. EDC (0.065 g), NHS (0.039 g) and oleic acid (0.3 mL) were added to 80 mL methanol, and the reaction mixture stirred for 15 min. The mixture was added dropwise to a solution made of 1 g of chitosan oligosaccharide dissolved in 100 mL of 1% acetic acid. The resulting solution stirred at room temperature for 48 h. The system was neutralized by NH_3_·H_2_O and then centrifuged at 1000×*g* for 5 min (Eppendorf 5810R, Germany). The precipitate was washed with methanol, dialyzed in distilled water for 48 h, dried under nitrogen and stored under argon at − 20 °C.

### Liposome preparation

To prepare CO-HPPH-TH302/Lipo, a lipid mixture of LPC, cholesterol, HPPH lipid, TH302 and CO-OA at a mass ratio of 20:2:2:6:3 was dissolved in chloroform and methanol at a volume ratio of 6:4 and then dried with a rotary evaporator at 56 °C. Afterwards, the dried lipid film was hydrated with 10 mL of phosphate buffered saline (PBS) and stirred at 56 °C for 60 min. The unencapsulated TH302, HPPH and CO-OA were removed via Millipore (Merck, Germany) with a molecular weight cut off (MWCO) of 100 kDa. The other liposomes, free-Liposome, CO-Liposome, TH302/Lipo, CO-TH302/Lipo, HPPH/Lipo, CO-HPPH/Lip and HPPH-TH302/Lipo were prepared by the same protocol.

### Characterization of the liposomes

The morphologies of the liposomes were observed by transmission electron microscopy (TEM, Hitachi, Tokyo, Japan) after being stained with phosphotungstic acid (1 wt%). The liposome size in water and zeta potentials were determined using Zeta-Plus analyzer (Brookhaven Instruments Co., Holtsville, NY, USA). The phase transition temperature was detected by differential scanning calorimetry (DSC, Netzsch, Germany). The entrapment rate of TH302 and HPPH was quantified by using ultraviolet–visible spectrophotometry (UV–VIS spectrophotometry, UV-3600, Shimadzu, Tokyo, Japan) at absorbances of 331 nm and 660 nm. The absorbance spectra of the different kinds of liposomes were recorded by ultraviolet–visible spectrophotometry.

### Evaluation of the singlet oxygen generation ability

The various liposomes were prepared at a final HPPH concentration of 0.025 mg/mL in PBS, mixed with the probe singlet oxygen sensor green (SOSG) at a final concentration of 2.5 μM and detected with an F-2700 fluorescence spectrophotometer (Hitachi, Japan) every 1 min for the first 5 min, then every 5 min for another 25 min. The various liposomes’ electron spin resonance (ESR) spectra were detected by the electron paramagnetic resonance spectrometer (Bruker, E500-9.5/12, Germany) before and after 10 min, 660 nm light irradiation. MDA-MB-231 cells were incubated with DCFH-DA at a final concentration of 10 µmol/L in RPMI 1640 for 20 min, washed 3 times with PBS, incubated with various liposomes at a final HPPH concentration of 0.025 mg/mL for 20 min, irradiated by a 660 nm laser for 10 min and finally detected with an F-2700 fluorescence spectrophotometer.

### IC50 values for TH302 and HPPH

MDA-MB-231 cells were treated with TH302 at final concentrations of 12.5 mg/L, 25 mg/L, 50 mg/L, 100 mg/L, 200 mg/L, 300 mg/L, and 400 mg/L. Then, the cells were maintained at 37 °C with 5% CO_2_ and 1% O_2_ in a humidified incubator as the hypoxic group and maintained at 37 °C with 5% CO_2_ in a humidified incubator as the normoxic group. MDA-MB-231 cells were incubated with HPPH at final concentrations of 12.5 mg/L, 25 mg/L, 50 mg/L, 100 mg/L, 200 mg/L, 300 mg/L, and 400 mg/L for 2 h. Then, the cells were either irradiated by a 660 nm laser for 15 min in the ‘with 660 nm’ group or not irradiated in the ‘without 660 nm’ group. The optical density (OD) values were examined by a cell counting kit-8 (CCK-8) assay. The viability rate of cell growth in each treatment group was calculated as follows: viability rate = [(OD in experimental group − OD in blank group)/(OD in control group − OD in blank group)] × 100%.

### Binding capacity of the CO-coated liposomes

To examine the binding capacity of the CO-coated liposomes, MDA-MB-231 cells and MCF-7 cells were divided into 5 groups as follows: (1) CO-FITC group; (2) HPPH/Lipo group; (3) CO-HPPH/Lipo group; (4) CO-FITC CD44-preblocked group; and (5) FITC-CO-TH302-HPPH/Lipo group. For confocal laser scanning microscopy (CLSM) observation, cells were seeded in laser confocal Petri dishes (Nest, Wuxi, China) at a density of 10^4^ cells per dish and incubated at 37 °C for 24 h. After that, the cells were incubated with CO-FITC, HPPH/Lipo, CO-HPPH/Lipo or FITC-CO-TH302-HPPH/Lipo for 2 h. For the pre-blocking groups, the cells were incubated with anti-CD44-PE for 20 min and then incubated with CO-FITC for another 2 h. Then, the cells were washed twice with PBS, fixed with 4% paraformaldehyde solution, stained with 4,6-diamino-2-phenyl indole (DAPI), and finally observed by CLSM (Olympus, Japan). For flow cytometry, cells were seeded in 6 well cell culture plates. (Corning, China) at a density of 10^5^ cells per well and incubated at 37 °C for 24 h. Free-MDA-MB-231 cells as the control group, liposomes with CO co-incubation with MDA-MB-231 cells as the positive group, liposomes without CO co-incubation with MDA-MB-231 cells as the negative group, liposomes with CO co-incubation with CD44-preblocked MDA-MB-231 cells. The fluorescence intensity was detected by flow cytometry (ACEA NovoCyte, ACEA Biosciences, USA).

### Fluorescence imaging in vitro

For fluorescence imaging, tumor-bearing mice were anesthetized and injected with CO-HPPH-TH302/Lipo and HPPH-TH302/Lipo at a dose of 5 mg/kg (concentration of HPPH), respectively. Then, at different time intervals p.i., the mice were imaged with a Maestro in vivo optical imaging system (Cambridge Research & Instrumentation, Inc.).

### Cytotoxicity of different liposomes in vitro

To determine if the target therapy could effectively inhibit TNBC, cells were divided into nine groups: (i) PBS group; (ii) free-Liposome group; (iii) CO-Liposome group; (iv) TH302/Lipo group; (v) CO-TH302/Lipo group; (vi) HPPH/Lipo group; (vii) CO-HPPH/Lipo group; (viii) HPPH-TH302/Lipo group; and (ix) CO-HPPH-TH302/Lipo group. The concentration of TH302 was 0.1 mg/mL in each TH302-containing group, and the concentration of HPPH was 0.025 mg/mL in all HPPH-containing groups. The treatments with HPPH were irradiated by a 660 nm light at 50 mW/cm^2^ for 15 min. The optical density (OD) values were examined by a cell counting kit-8 (CCK-8) assay. The viability rate of cell growth in each treatment group was calculated as follows: viability rate = [(OD in experimental group − OD in blank group)/(OD in control group − OD in blank group)] × 100%.

### Animals and tumor models

BALB/c nude mice (female, aged 5 weeks) were purchased from the Nanjing Biomedical Research Institute of Nanjing University (Jiangsu, China). The human cell line and animal studies were approved by the ethics committee of Southeast University, Nanjing, People’s Republic of China. All animals received humane care in compliance with the Principles of Laboratory Animal Care formulated by the National Society for Medical Research. To build the MDA-MB-231 tumor model, 2 × 10^6^ MDA-MB-231 cells in 100 μL of PBS were injected into the mammary fat pad of each mouse. The mice were used when the tumors grew to a volume of approximately 100 mm^3^.

### Fluorescence imaging in vivo

For fluorescence imaging, tumor-bearing mice were anesthetized and injected with CO-HPPH-TH302/liposomes at a dose of 5 mg/kg (concentration of HPPH). Then, at different time intervals p.i., the mice were imaged with a Maestro in vivo optical imaging system (Cambridge Research & Instrumentation, Inc.) with excitation at 660 nm.

### Treatment of liposomes in vivo

Fifty-four tumor-bearing mice were randomly divided into 9 groups for the MDA-MB-231 tumor model as follows: (i) saline group; (ii) free-Liposome group; (iii) CO-Liposome group; (iv) TH302/Lipo group; (v) CO-TH302/Lipo group; (vi) HPPH/Lipo group; (vii) CO-HPPH/Lipo group; (viii) HPPH-TH302/Lipo group; and (ix) CO-HPPH-TH302/Lipo group. The dose of TH302 was 30 mg/kg, and the concentration of HPPH was 5 mg/kg. Each treatment was injected via the tail vein, and 24 h later, the tumor region of each mouse treated with HPPH was exposed to a 660 nm light at 200 mW/cm^2^ for 15 min. The treatment was performed only once. The length (L) and width (W) of the tumor were measured from the beginning of the treatment until 15 days after treatment. The tumor volume (V) was calculated in terms of the formula: V = L × W × W/2. Fifteen days after treatment, the mice were sacrificed, and the tumor were collected.

The biosafety was evaluated by the main organs, including the heart, lung, spleen, kidney and liver, collected from each group for H&E staining 15 days post-treatment. The body weight of the tumor-bearing nude mice was measured every other day for 30 days after treatment.

To examine the antitumor effect of each group, one mouse was sacrificed from each group 1 day after irradiation, and the tumors were excised for H&E staining.

### Statistical analysis

Statistical analysis was performed using the GraphPad Prism version 7.0 software (GraphPad Software, Inc., San Diego, CA, USA) and ImageJ software (National Institutes of Health, USA). The differences between two groups were considered statistically significant for *P < 0.05, and very significant for **P < 0.01, ***P < 0.001.

## Supplementary Information

**Additional file 1: Figure S1.** The TLC results of OA-HNS and CO-OA. (A) TLC results of OA-NHS. (B) TLC results of CO-OA. **Figure S2.** HPLC–MS analysis of HPPH lipid. **Figure S3.** The surface potentials of HPPH-TH302/Lipo and CO-HPPH-TH302/Lipo. (A) Zeta potential of HPPH-TH302/Lipo; (B) Zeta potential of CO-HPPH-TH302/Lipo; (C) Zeta potentials of HPPH-TH302/Lipo and CO-HPPH-TH302/Lipo, each bar represents the mean ± SD of five replicates. **Figure S4.** Singlet oxygen production before 660 nm irradiation was measured by electron spin resonance (ESR). (A) Liposome group; (B) CO-Liposome; (C) TH302/Lipo; (D) CO-TH302/Liposome; (E) HPPH/Lipo; (F) CO-HPPH/Lipo; (G) HPPH-TH302/Lipo; (H) CO-HPPH-TH302/Lipo. **Figure S5.** Singlet oxygen production after 660 nm irradiation was measured by electron spin resonance (ESR). (A) Liposome group; (B) CO-Liposome; (C) TH302/Lipo; (D) CO-TH302/Liposome; (E) HPPH/Lipo; (F) CO-HPPH/Lipo; (G) HPPH-TH302/Lipo; (H) CO-HPPH-TH302/Lipo. **Figure S6.** The fluorescence signal between targeted and non-targeted liposomes detected by flow cytometry. Flow cytometric analysis of FITC positive MDA-MB-231 cells: (i) Control group: free-MDA-MB-231 cells; (ii) CO-Lipo group: liposomes with CO co-incubation with MDA-MB-231 cells; (iii) Lipo group: liposomes without CO co-incubation with MDA-MB-231 cells; (iv) Blocked CO-Lipo group: liposomes with CO co-incubation with CD44-preblocked MDA-MB-231 cells. **Figure S7.** The cytotoxicity of various liposomes without HPPH that illuminated by 660 nm LED in vitro. (A) Inhibition of viability of MDA-MB-231 cells; (B) Inhibition of viability of MCF-7 cells, each bar represents the mean ± SD of five replicates.

## Data Availability

The datasets used and analyzed during the current study are available from the corresponding author on reasonable request.

## Abbreviations

TNBC: Triple negative breast cancer
CD44: Cluster of differentiation-44
CO: Chitosan oligosaccharide
HPPH: Photochlor
DSC: Differential scanning calorimetry
PDT: Photodynamic therapy
ERs: Estrogen receptors
PRs: Progesterone receptors
HERs: Human epidermal growth factor receptors
BIDC: Breast invasive ductal carcinoma
PSs: Photosensitizers
ROS: Reactive oxygen species
ESR: Electron spin resonance
MFIs: Mean fluorescence intensities
EPR: Enhanced permeability and retention effect
H&E: Hematoxylin and eosin
FDA: Food and Drug Administration
CCK-8: Cell counting kit-8
LPC: 1-Palmitoyl-2-hydroxy sn-glycero-3-phosphocholine
OA: Oleic acid
EDC: *N*-(3-(Dimethylamino)-propyl)-*N*-ethylcarbodiimide hydrochloride crystalline
NHS: *N*-Hydroxysuccinimide
DIPEA: *N*,*N*-Diisopropylethylamine
DMAP: 4-(Dimethylamino) pyridine
FBS: Fetal bovine serum
PBS: Phosphate buffered saline
MWCO: Molecular weight cut off
TEM: Transmission electron microscopy
UV–VIS: Ultraviolet–visible
OD: Optical density
CLSM: Confocal laser scanning microscopy
DAPI: 4,6-Diamino-2-phenyl indole
MFIs: Mean fluorescence intensities
HPLC: High performance liquid chromatography
MS: Mass spectrum
