# Correction: Chitosan oligosaccharide decorated liposomes combined with TH302 for photodynamic therapy in triple negative breast cancer

**DOI:** 10.1186/s12951-025-03268-3

**Published:** 2025-04-05

**Authors:** Yinan Ding, Rui Yang, Weiping Yu, Chunmei Hu, Zhiyuan Zhang, Dongfang Liu, Yanli An, Xihui Wang, Chen He, Peidang Liu, Qiusha Tang, Daozhen Chen

**Affiliations:** 1 Medical School of Southeast University, Nanjing, 210009 China; 2 Research Institute for Reproductive Health and Genetic Diseases, The Affiliated Wuxi Maternity and Child Health Care Hospital of Nanjing Medical University, Wuxi, 214002 China; 3 Department of Tuberculosis, The Second Affiliated Hospital of Southeast University (The Second Hospital of Nanjing), Nanjing, 210009 China; 4 Department of Neurosurgery, Nanjing Jinling Hospital, Nanjing University, Nanjing, 210002 China; 5 Afliated Zhongda Hospital of Southeast University, Nanjing, 210009 China

**Correction to: J Nanobiotechnol (2021) 19:147.** 10.1186/s12951-021-00891-8

In this article, the photosensitizer “HPPH” was inaccurately named as "pyro-acid” in the Graphical Abstract, Scheme 1 and Scheme 2.

Incorrect Graphical Abstract:


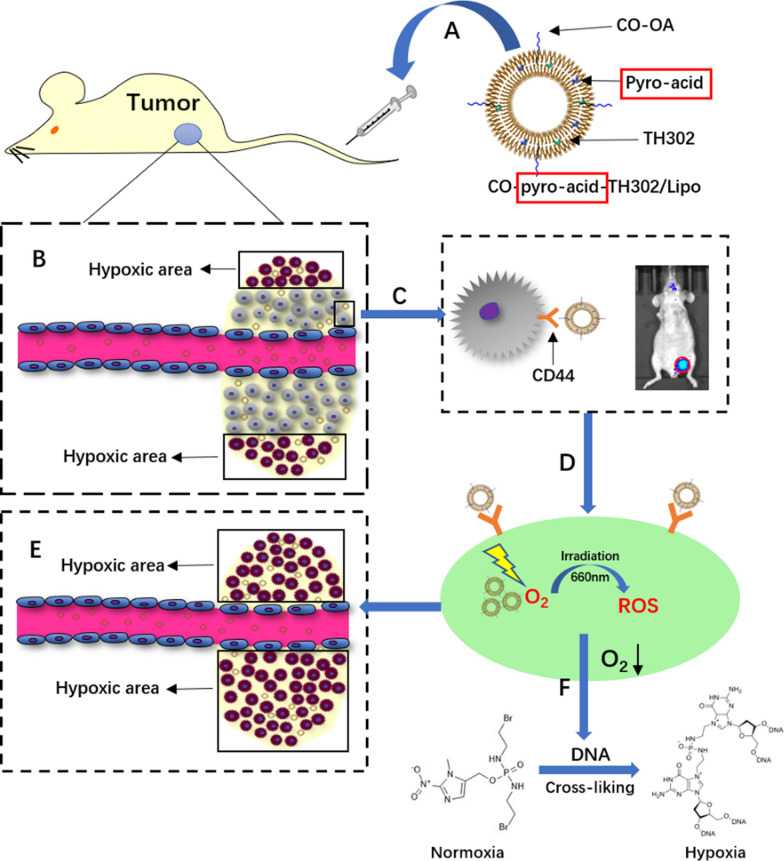


Correct Graphical Abstract:


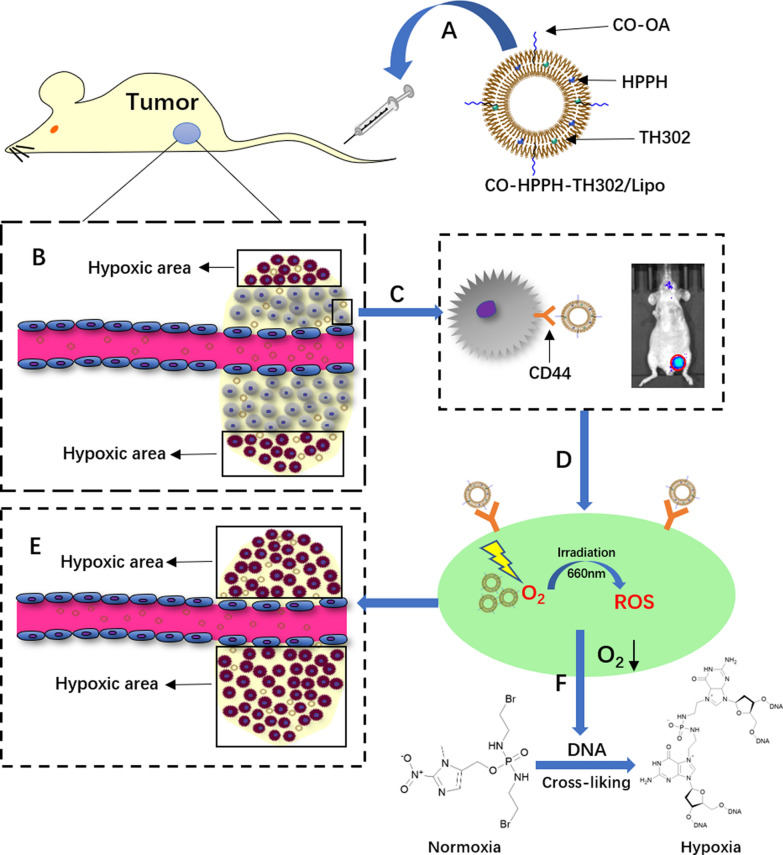


Incorrect Scheme 1:


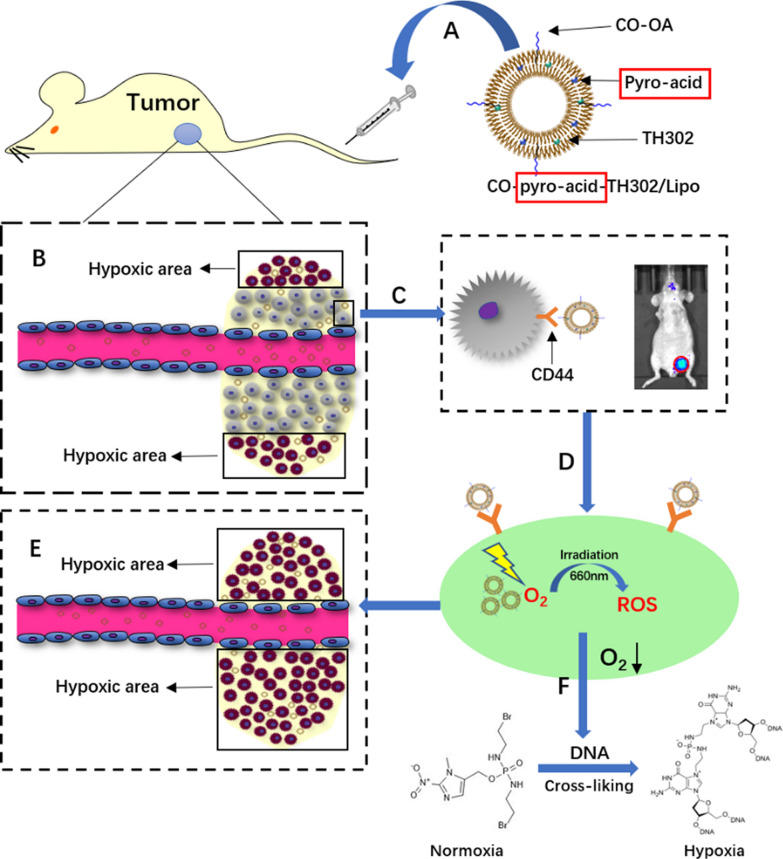


Correct Scheme 1:


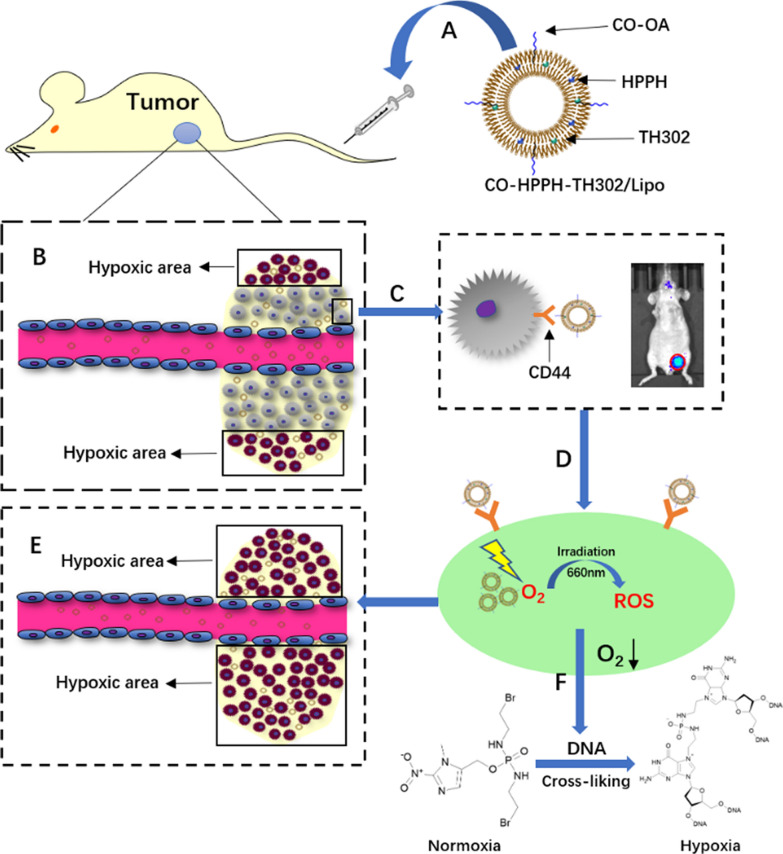


Incorrect Scheme 2:


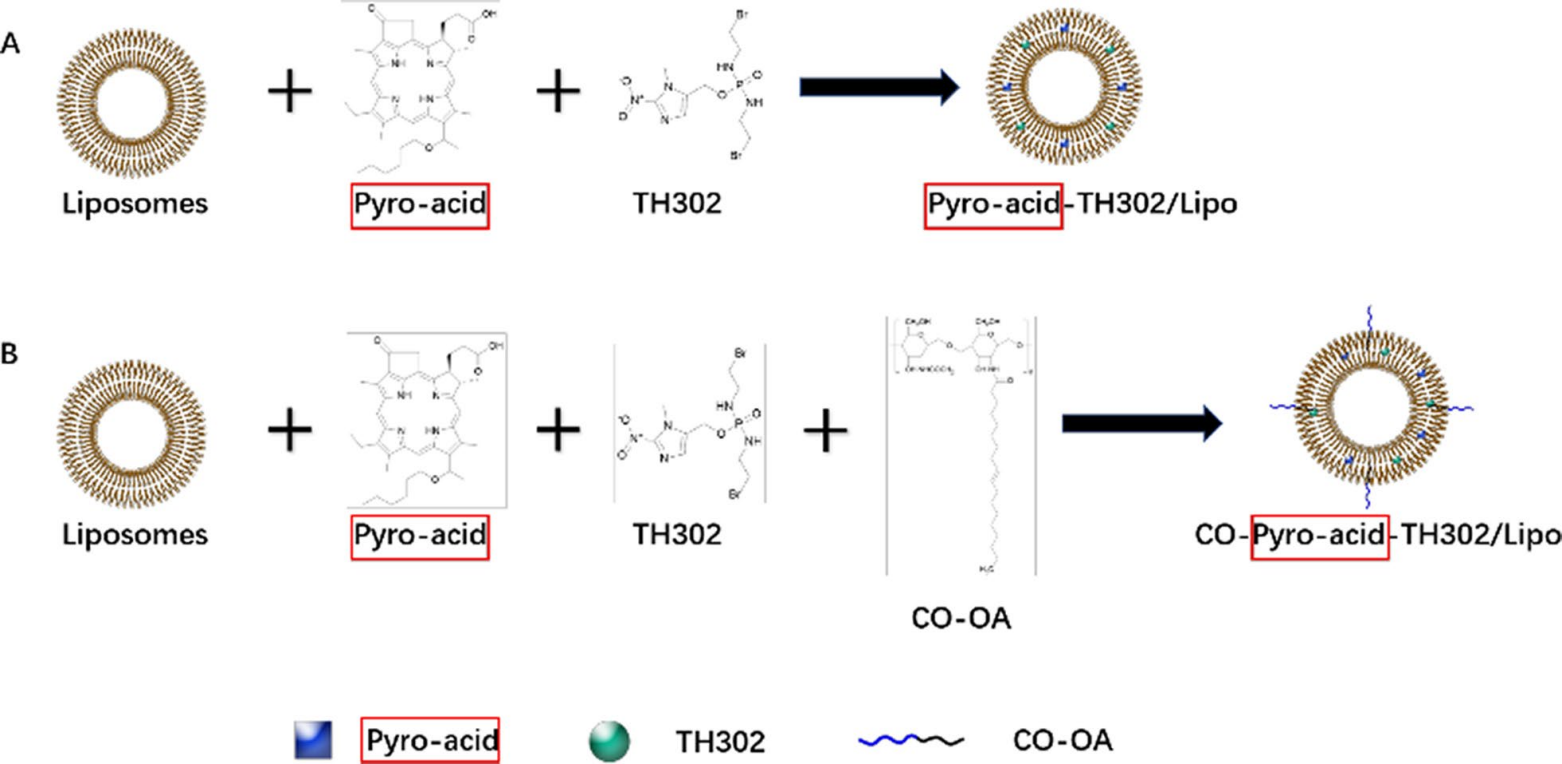


Corrected Scheme 2:


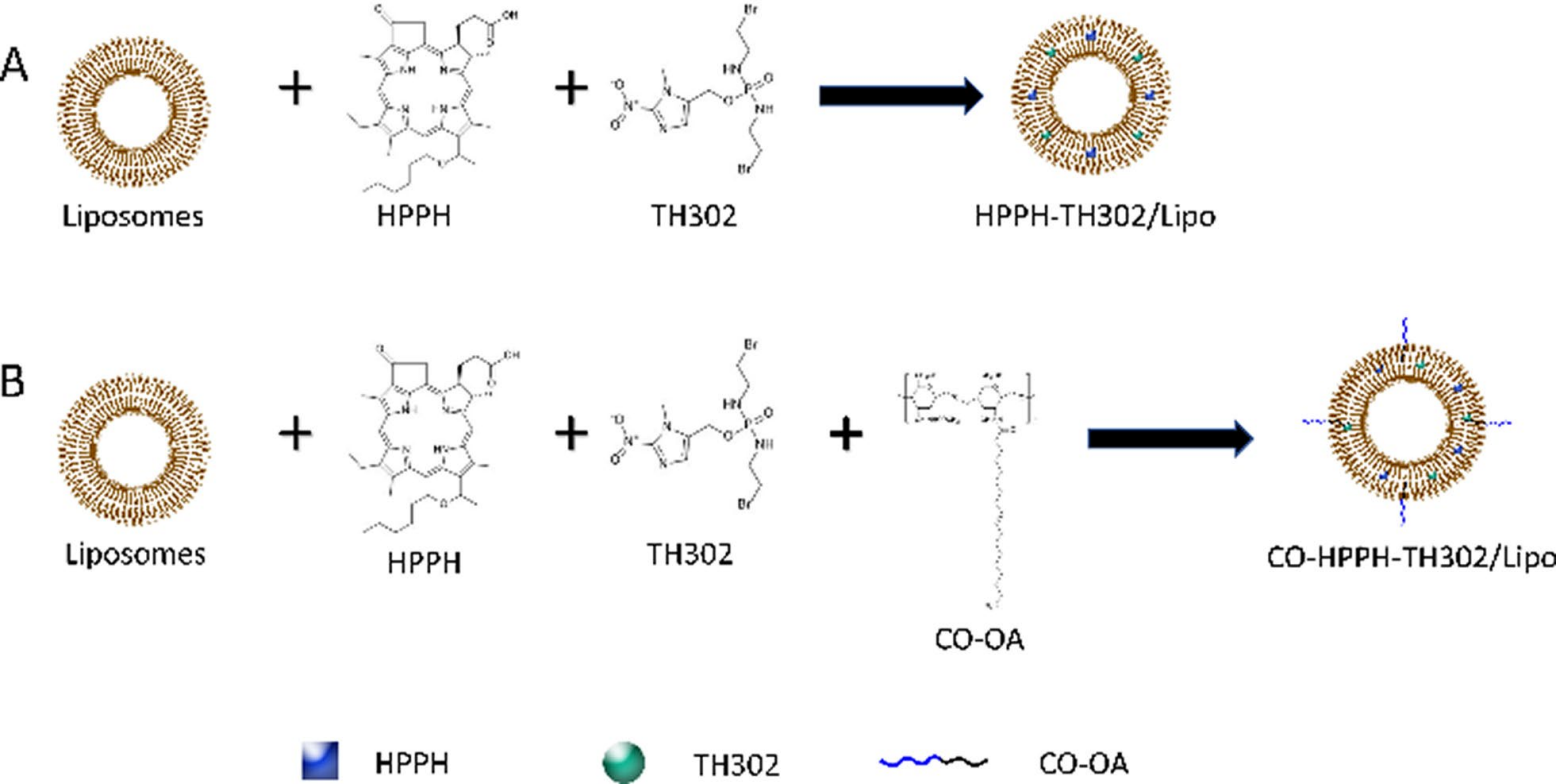


In Fig. 7c, the HE staining images for the CO-Liposome group, CO-TH302/Lipo group, CO-HPPH/Lipo group in the spleen, and the CO-Liposome group in the kidney did not correspond to the correct groups. The authors sincerely apologize for these mistakes.

Incorrect Fig. 7:


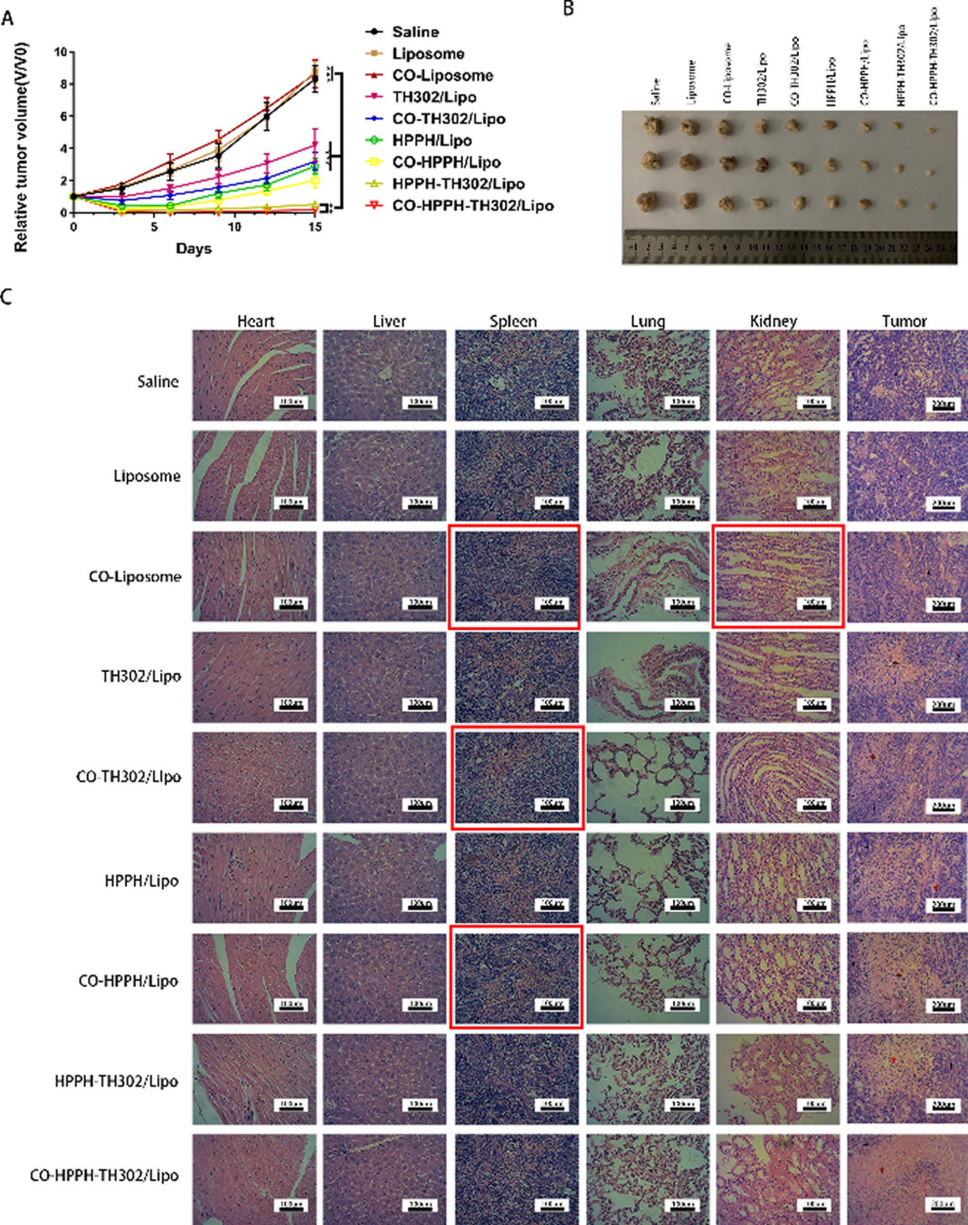


Correct Fig. 7:


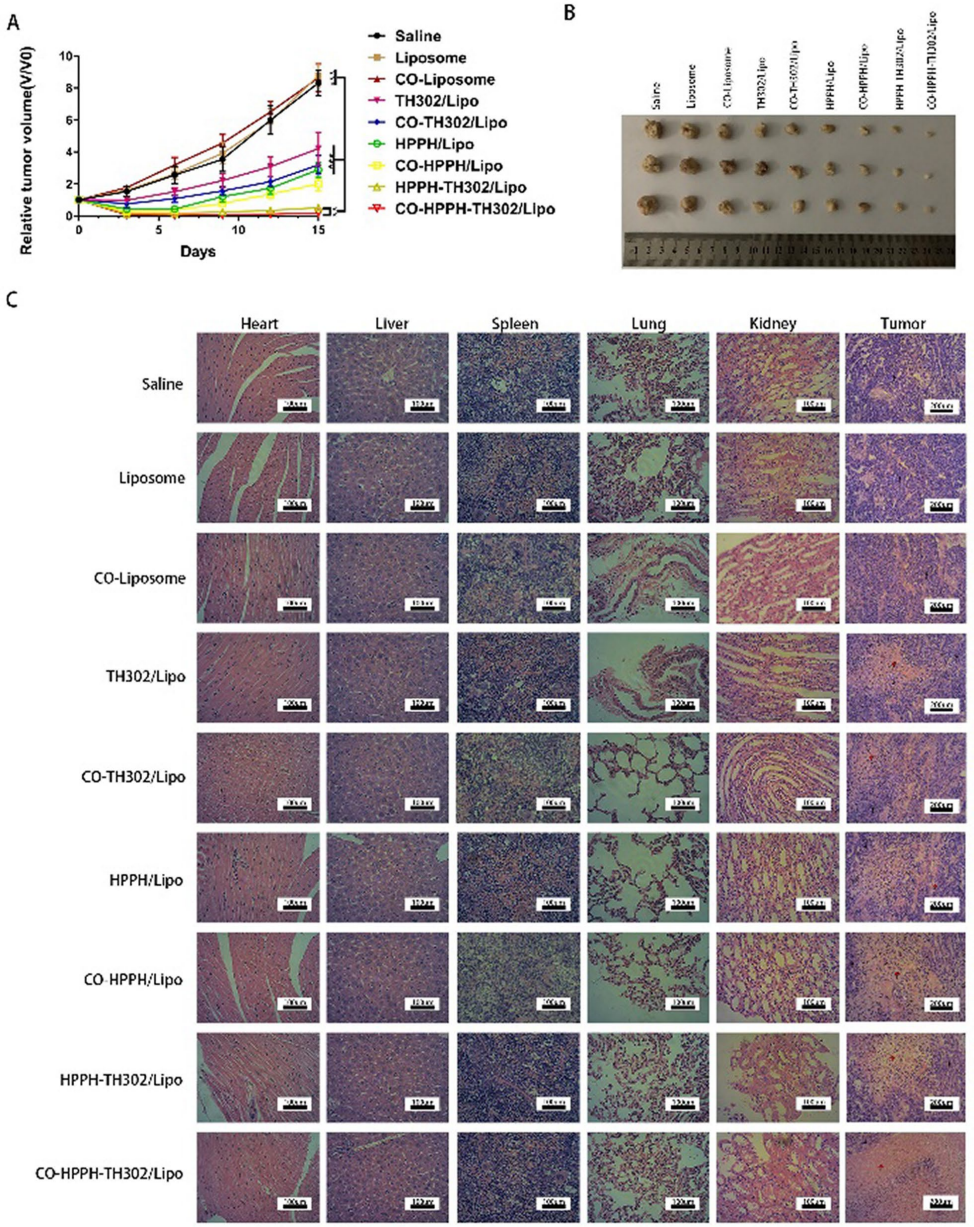


The correct Graphical Abstract, Scheme 1, Scheme 2 and Fig. 7 have been included in this Correction, and the original article has been corrected.

