# Supplementary material for: Chitosan oligosaccharide decorated liposomes combined with TH302 for photodynamic therapy in triple negative breast cancer
Source: J Nanobiotechnology. 2021 May 19;19:147. doi: 10.1186/s12951-021-00891-8 (PMC8136194; doi:10.1186/s12951-021-00891-8)
Supplement: Supplementary file 1 — Additional file 1: Figure S1. The TLC results of OA-HNS and CO-OA. (A) TLC results of OA-NHS. (B) TLC results of CO-OA. Figure S2. HPLC–MS analysis of HPPH lipid. Figure S3. The surface potentials of HPPH-TH302/Lipo and CO-HPPH-TH302/Lipo. (A) Zeta potential of HPPH-TH302/Lipo; (B) Zeta potential of CO-HPPH-TH302/Lipo; (C) Zeta potentials of HPPH-TH302/Lipo and CO-HPPH-TH302/Lipo, each bar represents the mean ± SD of five replicates. Figure S4. Singlet oxygen production before 660 nm irradiation was measured by electron spin resonance (ESR). (A) Liposome group; (B) CO-Liposome; (C) TH302/Lipo; (D) CO-TH302/Liposome; (E) HPPH/Lipo; (F) CO-HPPH/Lipo; (G) HPPH-TH302/Lipo; (H) CO-HPPH-TH302/Lipo. Figure S5. Singlet oxygen production after 660 nm irradiation was measured by electron spin resonance (ESR). (A) Liposome group; (B) CO-Liposome; (C) TH302/Lipo; (D) CO-TH302/Liposome; (E) HPPH/Lipo; (F) CO-HPPH/Lipo; (G) HPPH-TH302/Lipo; (H) CO-HPPH-TH302/Lipo. Figure S6. The fluorescence signal between targeted and non-targeted liposomes detected by flow cytometry. Flow cytometric analysis of FITC positive MDA-MB-231 cells: (i) Control group: free-MDA-MB-231 cells; (ii) CO-Lipo group: liposomes with CO co-incubation with MDA-MB-231 cells; (iii) Lipo group: liposomes without CO co-incubation with MDA-MB-231 cells; (iv) Blocked CO-Lipo group: liposomes with CO co-incubation with CD44-preblocked MDA-MB-231 cells. Figure S7. The cytotoxicity of various liposomes without HPPH that illuminated by 660 nm LED in vitro. (A) Inhibition of viability of MDA-MB-231 cells; (B) Inhibition of viability of MCF-7 cells, each bar represents the mean ± SD of five replicates. [file 12951_2021_891_MOESM1_ESM.docx]

Chitosan Oligosaccharide Decorated Liposomes Combined with TH302 for Photodynamic Therapy in Triple Negative Breast Cancer

Yinan Ding^1^, Rui Yang^2^, Weiping Yu^1^, Chunmei Hu^3^, Zhiyuan Zhang^4^, Dongfang Liu^1^, Yanli An^5^, Xihui Wang^1^, Chen He^1^, Peidang Liu^1^, Qiusha Tang^1^* and Daozhen Chen^2^*

^1^Medical School of Southeast University, Nanjing 210009, China

^2^Research Institute for Reproductive Health and Genetic Diseases, The Affiliated Wuxi Maternity and Child Health Care Hospital of Nanjing Medical University, Wuxi, 214002, China

^3^Department of Tuberculosis, the Second Affiliated Hospital of Southeast University (the second hospital of Nanjing), Nanjing 210009, China

^4^Department of Neurosurgery, Nanjing Jinling hospital, Nanjing University, Nanjing 210002, China

^5^Affiliated Zhongda Hospital of Southeast University, Nanjing 210009, China

*Correspondence: panyixi-tqs@163.com, chendaozhen@163.com

**
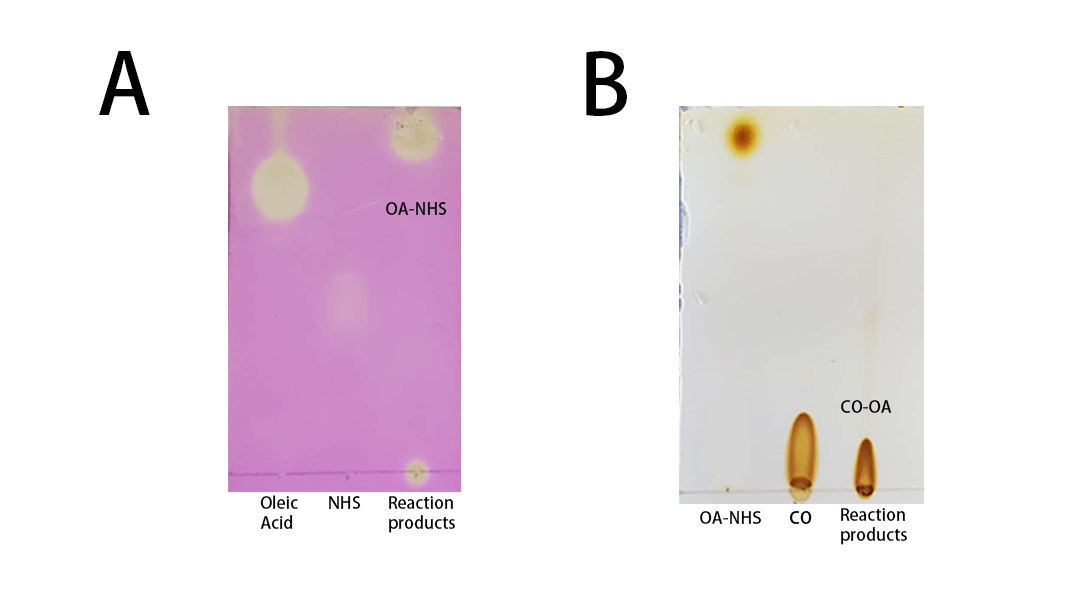
**

**Figure S1** The TLC results of OA-HNS and CO-OA. (A) TLC results of OA-NHS. (B) TLC results of CO-OA.


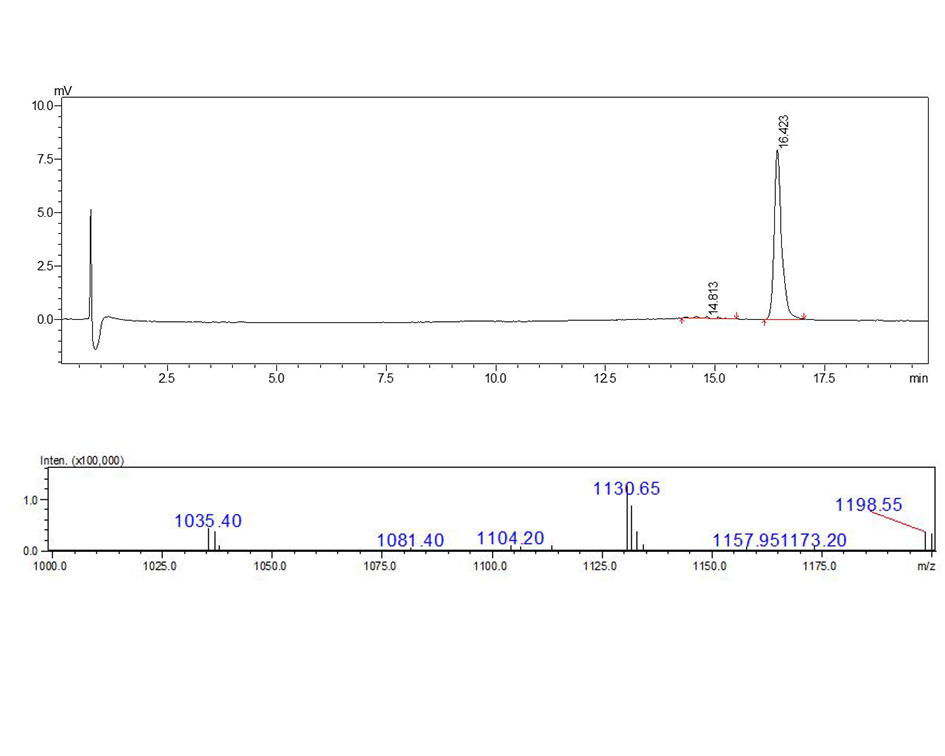


**Figure S2** HPLC-MS analysis of HPPH lipid.


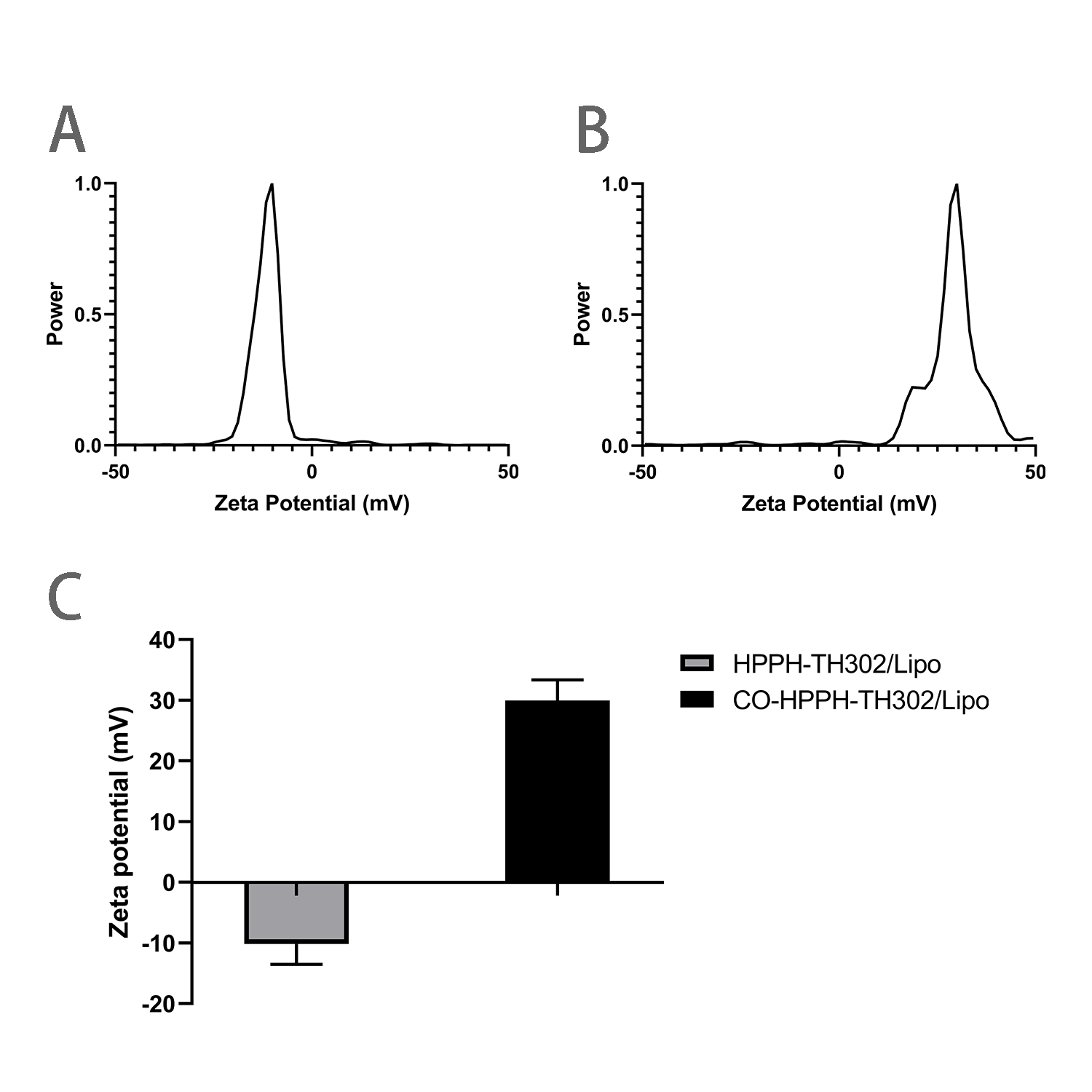


**Figure S3.** The surface potentials of HPPH-TH302/Lipo and CO-HPPH-TH302/Lipo. (A) Zeta potential of HPPH-TH302/Lipo; (B) Zeta potential of CO-HPPH-TH302/Lipo; (C) Zeta potentials of HPPH-TH302/Lipo and CO-HPPH-TH302/Lipo, each bar represents the mean ± SD of five replicates.


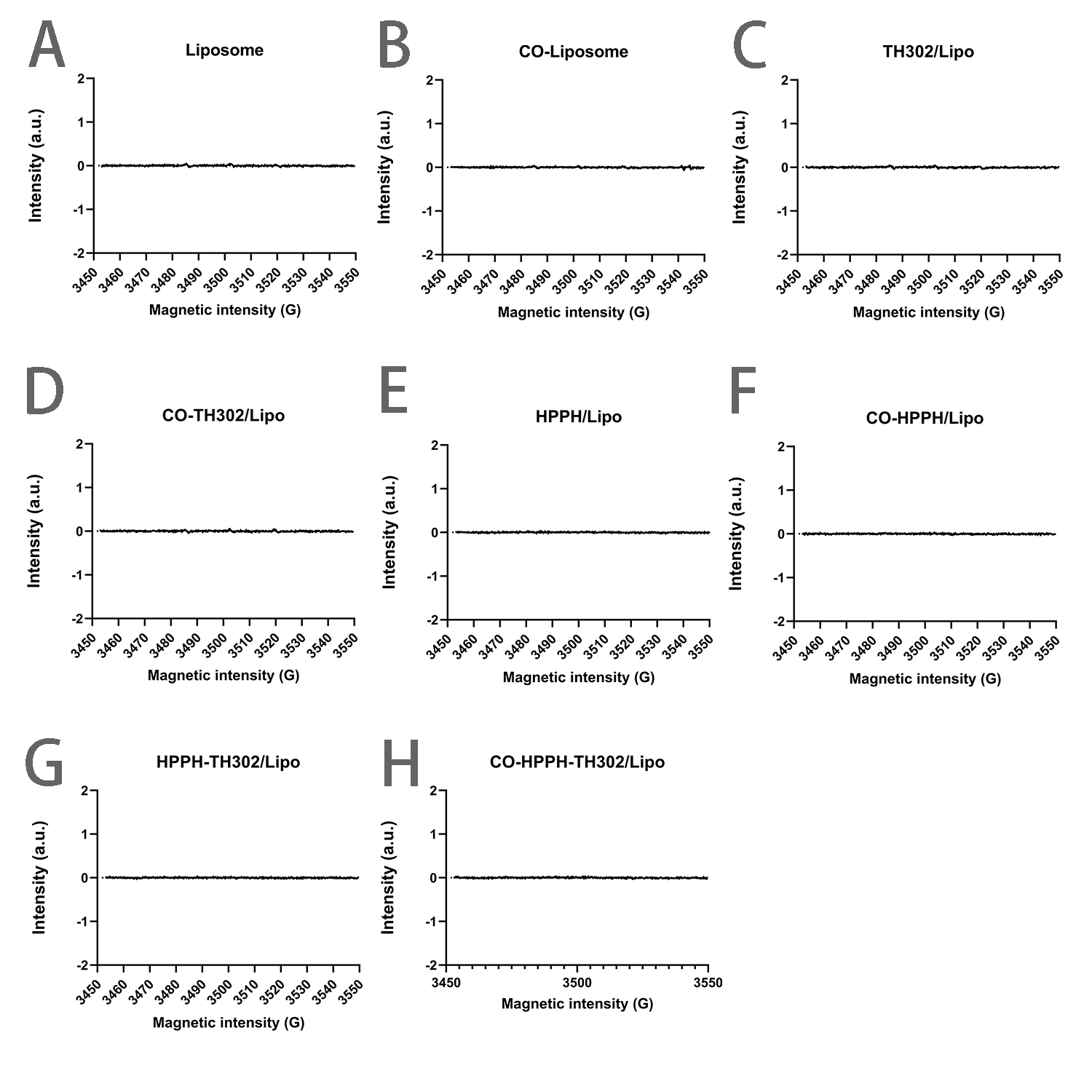


**Figure S4.** Singlet oxygen production before 660nm irradiation was measured by electron spin resonance (ESR). (A) Liposome group; (B) CO-Liposome; (C) TH302/Lipo; (D) CO-TH302/Liposome; (E) HPPH/Lipo; (F) CO-HPPH/Lipo; (G) HPPH-TH302/Lipo; (H) CO-HPPH-TH302/Lipo.


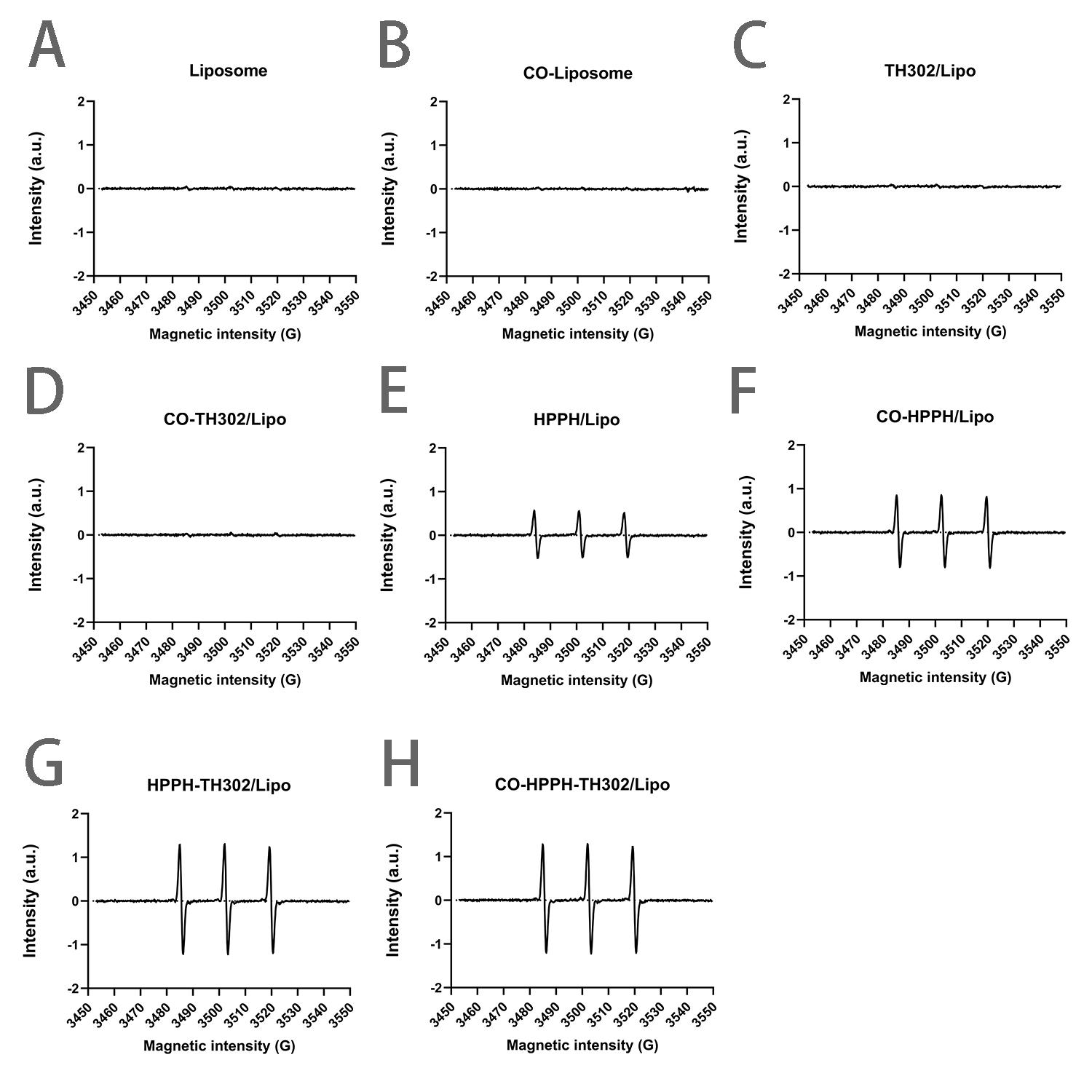


**Figure S5.** Singlet oxygen production after 660nm irradiation was measured by electron spin resonance (ESR). (A) Liposome group; (B) CO-Liposome; (C) TH302/Lipo; (D) CO-TH302/Liposome; (E) HPPH/Lipo; (F) CO-HPPH/Lipo; (G) HPPH-TH302/Lipo; (H) CO-HPPH-TH302/Lipo.


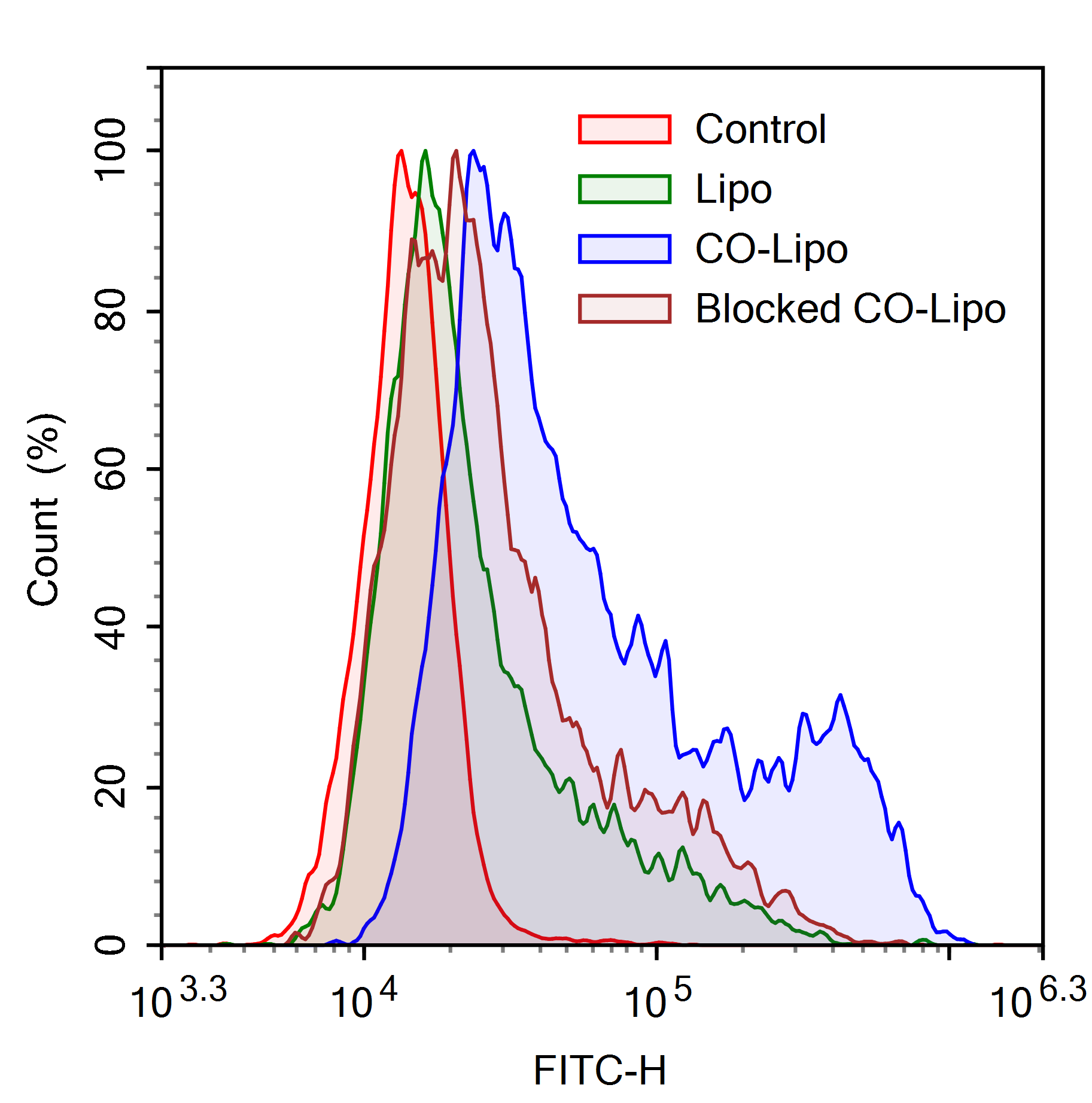


**Figure S6.** The fluorescence signal between targeted and non-targeted liposomes detected by flow cytometry. Flow cytometric analysis of FITC positive MDA-MB-231 cells: i) Control group: free-MDA-MB-231 cells; ii) CO-Lipo group: liposomes with CO co-incubation with MDA-MB-231 cells; iii) Lipo group: liposomes without CO co-incubation with MDA-MB-231 cells; iv) Blocked CO-Lipo group: liposomes with CO co-incubation with CD44-preblocked MDA-MB-231 cells.

**
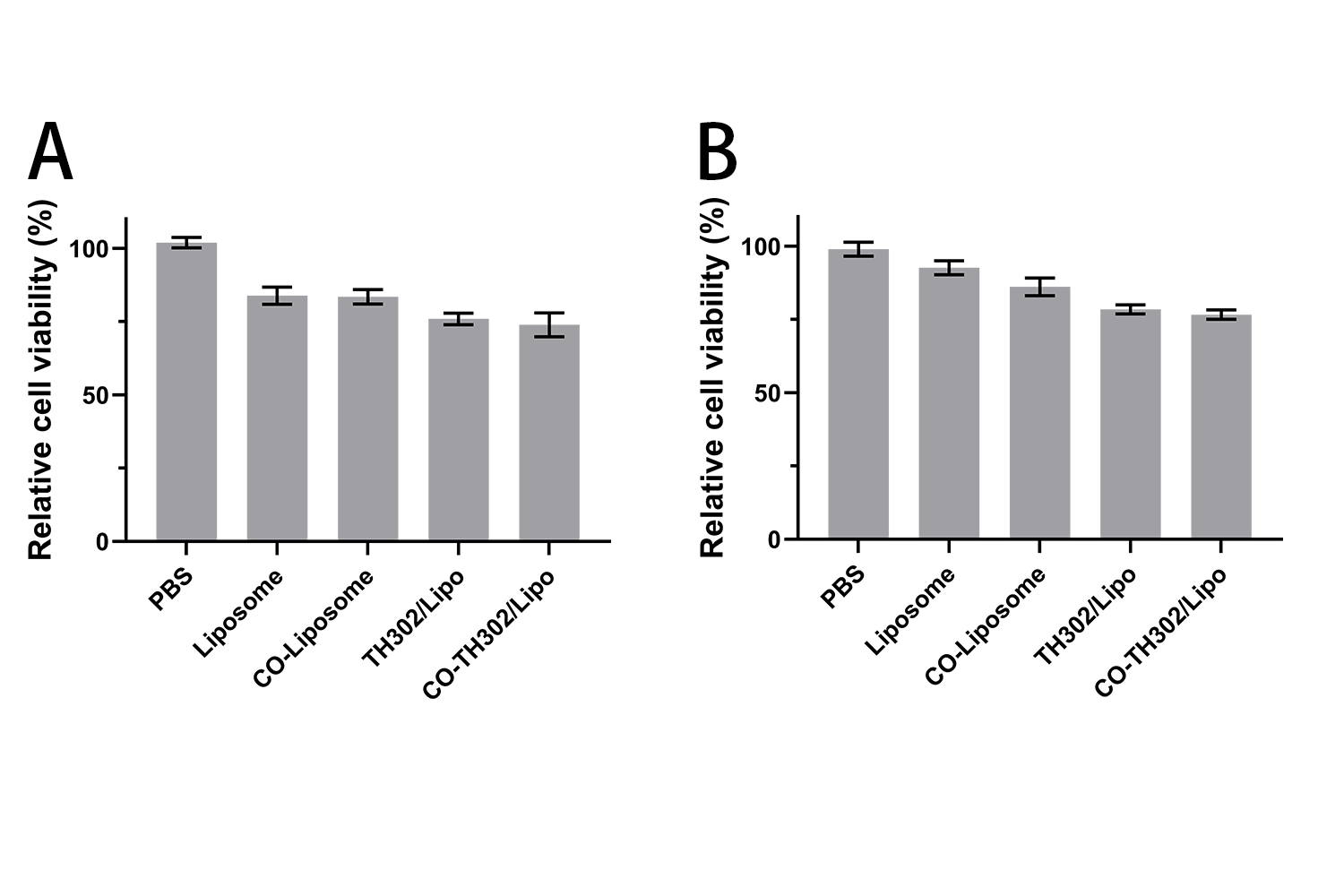
**

**Figure S7.** The cytotoxicity of various liposomes without HPPH that illuminated by 660nm LED in vitro. (A) Inhibition of viability of MDA-MB-231 cells; (B) Inhibition of viability of MCF-7 cells, each bar represents the mean ± SD of five replicates.


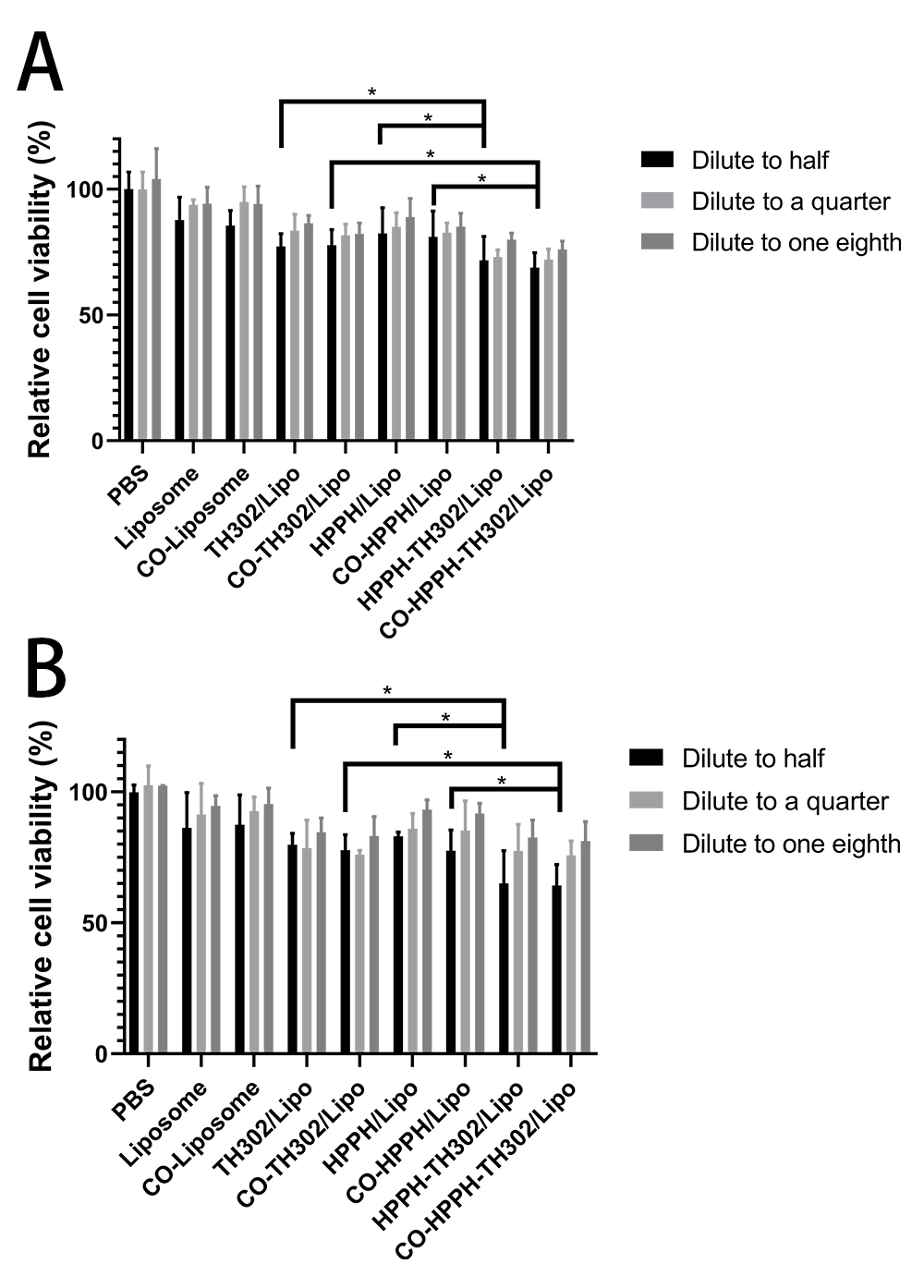


**Figure S8.** The cytotoxicity of gradient diluted liposomes in (A) MDA-MB-231 and (B) MCF-7, each bar represents the mean ± SD of five replicates. * means P < 0.05, ** means P < 0.01, and *** means P < 0.001.


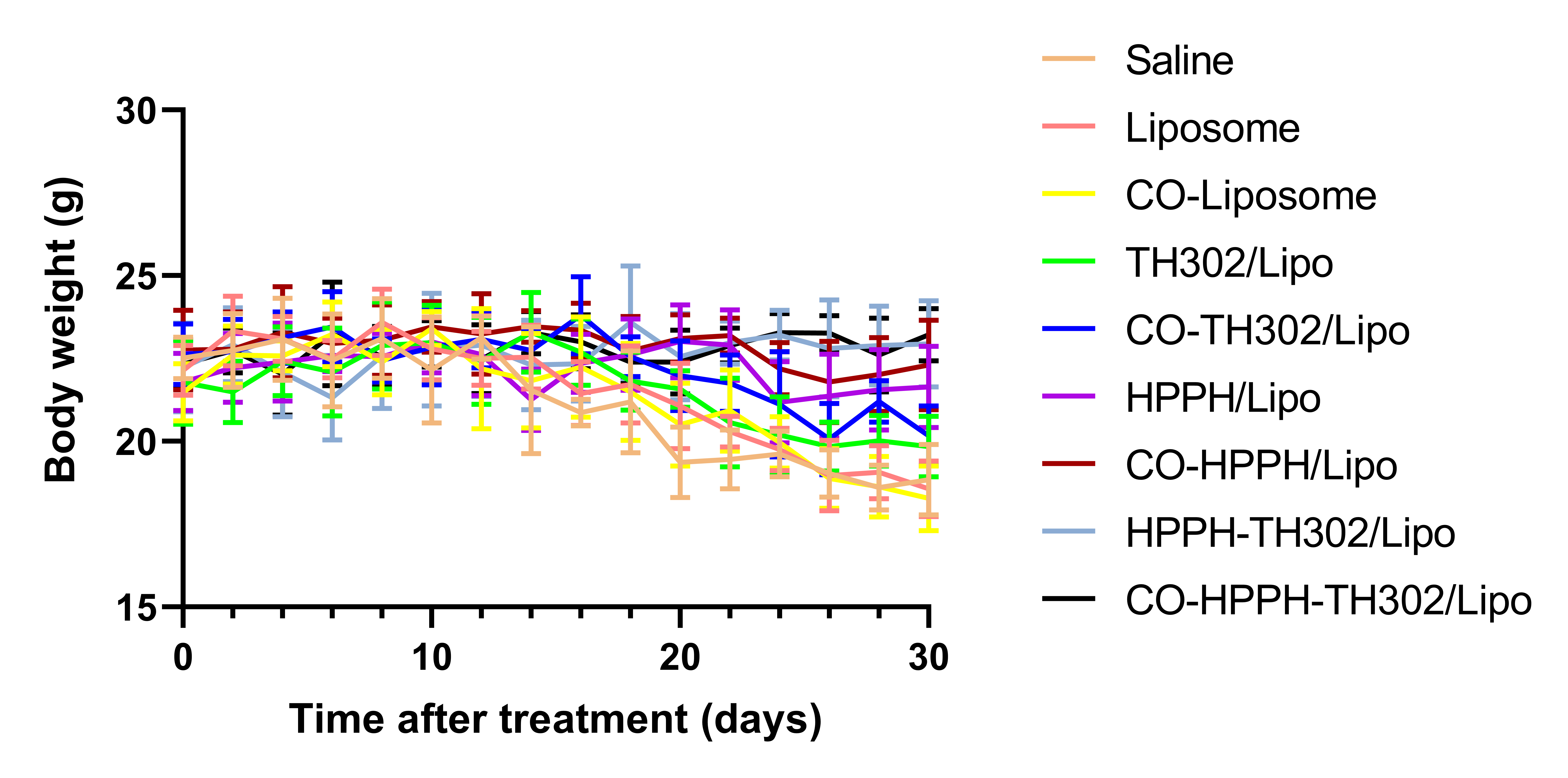


**Figure S9** The body weight of tumor-bearing nude mice in each group after treatment.
